# Integrins regulate stemness in solid tumor: an emerging therapeutic target

**DOI:** 10.1186/s13045-021-01192-1

**Published:** 2021-10-29

**Authors:** Jiangling Xiong, Lianlian Yan, Cheng Zou, Kai Wang, Mengjie Chen, Bin Xu, Zhipeng Zhou, Dingxiao Zhang

**Affiliations:** 1 School of Biomedical Sciences, Hunan University, Changsha, 410082 Hunan Province China; 2 College of Biology, Hunan University, Changsha, 410082 Hunan Province China; 3 Department of Urology, School of Medicine, Affiliated Zhongda Hospital of Southeast University, Nanjing, 210009 Jiangsu Province China; 4 College of Life Science and Technology, Huazhong Agricultural University, Wuhan, 430070 Hubei Province China

**Keywords:** Integrin, Stemness, Solid tumor, Cancer stem cells, Metastasis, Therapeutic targeting

## Abstract

Integrins are the adhesion molecules and transmembrane receptors that consist of α and β subunits. After binding to extracellular matrix components, integrins trigger intracellular signaling and regulate a wide spectrum of cellular functions, including cell survival, proliferation, differentiation and migration. Since the pattern of integrins expression is a key determinant of cell behavior in response to microenvironmental cues, deregulation of integrins caused by various mechanisms has been causally linked to cancer development and progression in several solid tumor types. In this review, we discuss the integrin signalosome with a highlight of a few key pro-oncogenic pathways elicited by integrins, and uncover the mutational and transcriptomic landscape of integrin-encoding genes across human cancers. In addition, we focus on the integrin-mediated control of cancer stem cell and tumor stemness in general, such as tumor initiation, epithelial plasticity, organotropic metastasis and drug resistance. With insights into how integrins contribute to the stem-like functions, we now gain better understanding of the integrin signalosome, which will greatly assist novel therapeutic development and more precise clinical decisions.

## Introduction

The term of integrin was first used to describe the receptor’s function of integrating the extracellular matrix (ECM) network to the cellular cytoskeletal network. As members of the membrane glycoprotein superfamily, integrins are transmembrane (TM) cell surface heterodimeric receptors consisting of an α and a β subunit. There are 18 α subunits and 8 β subunits in mammals, which to date form 24 αβ integrin heterodimers [1]. Importantly, each pair of heterodimers display both functional and tissue specificity involved in a plethora of biological processes in development and disease [2]. The assortment of integrin repertoire allows for adhesion to nearly all ECM components; and depending on different types of ligands, integrins can be classified mainly into four categories: (1) receptors that recognize the tripeptide RGD (Arg-Gly-Asp) sequence (including all five αv integrins, two β1 integrins (α5, α8) and αIIbβ3); (2) receptors that are leukocyte-specific and bind to LDV (L/I–D/E–V/S/T–P/S) sequences (including two α4 integrins (β1, β7), α9β1, αEβ7 and four members of β2 integrins); (3) receptors that bind selectively to laminin (including α6β4 and three β1 integrins (α3, α6, α7)); and (4) collagen-binding receptors (including four β1 integrins (α1, α2, α10, α11) recognizing G–F–O–G–E–R motif). Basically, the first category is RGD-dependent and the rest three are RGD-independent. Besides ECM molecules, integrins also bind to counter-receptors on the surface of neighboring cells, such as lg-superfamily cell surface receptor vascular cell adhesion molecule-1 (VCAM-1) and intercellular adhesion molecule-1 (ICAM-1) [3]. In general, through cognate ligand binding, integrins sense and trigger distinct intracellular signaling cascades in response to extracellular changes, which are frequently essential for physiological and pathological functions [4]. Notably, and interestingly, different categories of integrins can recognize and bind the same ligands, and the same integrins can bind to multiple distinct ligands [2], indicative of an intricate network of integrin signalosome. Here, we discuss the origins and consequences of deregulated integrin signaling in solid cancers, with an emphasis on their regulation in cancer stemness and therapeutic resistance. By summarizing the current situation of anti-integrin agents in pre-clinical and clinical practice, we also illustrate emerging mechanism-based therapeutic strategies to combat life-threatening cancers.

## Structure and working model of integrins

**Fig. 1:**
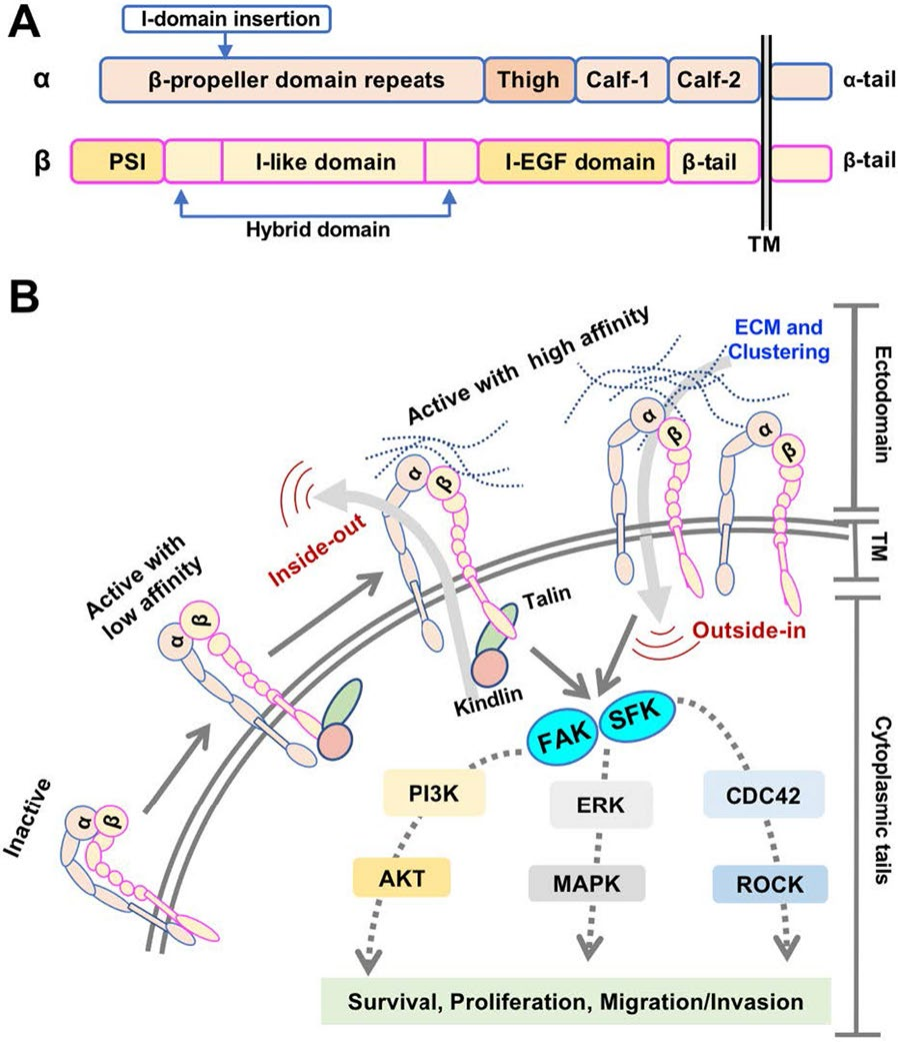
Integrin structure and integrin signalosome. **a** Schematic domain structure of a generic integrin. The α subunit contains 8 β-propeller domain repeats, a thigh domain, two calf domains (Calf-1 and Calf-2) and an α-tail domain. Notably, for 9 out of 18 α subunits, there is an I-domain inserted between β propeller domains 2 and 3. The β subunit typically comprises a plexin-semaphorin-integrin (PSI) domain, an I-like domain followed by a β-sandwich hybrid domain, four cysteine-rich integrin epidermal growth factor-like (I-EGF) repeats and a β-tail domain. **b** Bidirectional integrin signalosome. Integrins exist in different conformational states that determine the receptor affinity for ECM components and other ligands: from a bent-closed (inactive) to an extended closed (active with low affinity) and finally to an extended open conformation (active with high affinity). When binding to ECM proteins, integrins are activated and clustered, and are capable of eliciting downstream signaling and controlling cellular responses to environmental cues (outside-in signaling). Integrins also response to an “inside-out signaling”, whereby Talin binding to the β integrin tail triggers conformational switch to an extended open state and further recruit integrin activating proteins such as Kindlins to activate integrin. The most well-studied hub pathway activated by integrins is focal adhesion kinase (FAK), with subsequent recruitment and activation of the Src family kinase (SFK), which ultimately affects cell behavior via crosstalk with many other signaling effectors. See text for details

Despite the diversity of integrin heterodimers, integrins are evolutionally conserved in amino acid (aa) sequences and share a common structure (i.e., a large extracellular domain, a short transmembrane domain and a short cytoplasmic domain). The extracellular regions of both α and β subunits participate in ligand binding (Fig. 1a). Half of the 18 α-subunits (α1, α2, α10, α11, αD, αL, αM, αX and αE) have an additional 200aa I-domain inserted in the β-propeller domain [5]. The αI-domain possesses a metal ion-dependent adhesion site (MIDAS), the major binding site for ligands such as collagen and some laminins [6]. Similarly, the β subunit generally comprises of a plexin-sempahorin-integrin (PSI) domain, a hybrid domain (with an I-like domain, called βI, embedded in), an integrin epidermal growth factor-like (I-EGF) domain and a β-tail domain (Fig. 1a). The βI-domain is analogous to the αI-domain and participates in ligand binding by interacting with β-propeller domain in another 9 α-integrins without αI-domain (α3, α4, α5, α6, α7, α8, α9, αv and αIIb) [7]. The transmembrane helical domains (TMD) of the α and β subunits are conservative and an association between α-TMD and β-TMD directly correlates with integrin activation. Integrin cytoplasmic tails serve as nucleation center for integrin and intercellular protein interactions. Except the β4 tail which is approximately 1000aa long, the lengths of the α and β cytoplasmic tails are usually less than 75aa. Particularly, and unlike the other integrins, β4 integrin couples to the intermediate filament instead of actin cytoskeleton [8]. Most integrin β tails contain a NPxY/F motif, rendering binding to phosphotyrosine binding (PTB) domains-containing proteins such as Talin. Notably, the homology among β subunit cytoplasmic tails is strikingly high, whereas α tails are highly divergent except for a conserved GFFKR motif located next to the TM region.

It is widely accepted that integrin activation is a multi-step process accompanied with conformational changes. Structural studies have revealed that integrins have three conformation states (Fig. 1b): bent-closed (inactive), extended-closed (active with low affinity), and extended-open (active with high affinity) [4, 9]. In the inactive state, an integrin extracellular region is bent and the cytoplasmic tails of α and β subunits are closed together. The interaction between α and β tails further stabilizes the inactive conformation [9]. Upon binding to an adaptor protein Talin (a high molecular weight cytoskeletal protein concentrated at regions of cell-substratum contact) and/or Kindlin (an integrin-binding co-activator), β integrin tail is forced to separate, leading to an unfolding of the TM and extracellular domains that creates availability of a ligand binding pocket (active with low affinity) [4, 9]. Upon engaging the ECM, integrin straightens out and further separates the cytoplasmic tails. This conformational change permits the receptor to interact with cytoskeletal proteins and signal transducers (active with high affinity). In turn, these interactions further enhance the ligand-binding affinity and induce clustering of other activated integrins to facilitate strong focal adhesion formation [10]. This adhesion complexes connect intracellular cytoskeleton to the basement membrane by indirect integrin-actin connections (e.g., the α6β4 integrin associates with intermediate filament). Notably, mechanical forces assist reinforcement of the ECM-cytoskeleton link and recruitment of additional signaling proteins to activate integrin [11].

Integrins have no enzymatic activity and thus depend on binding to neighboring receptors and intracellular proteins to transmit mechanical and chemical signals to the cell interior and finally affect cellular functions. Approximately 150 adhesion proteins have been identified in part of integrin-mediated adhesion complex, which is known as focal adhesion or adhesome [4, 12]. Thus, the downstream signaling of an active integrin (i.e., integrin signalosome) is complex and cell specific, but typically involves autophosphorylation of the focal adhesion kinase (FAK) and subsequent recruitment and activation of Src family kinase (SFK) (Fig. 1b) [12]. FAK is a key signaling effector and can be activated by most integrins. It can interact directly, or indirectly through Talin and Paxillin, with the cytoplasmic tail of β subunits. FAK recruits the growth factor receptor bound protein 2 (GRB2) and actives Ras-ERK (extracellular signal regulated kinase)/MAPK (mitogen activated protein kinase) cascade to promote cell cycle progression and proliferation [13]. FAK can also interact with and activate phosphoinositide 3-OH kinase (PI3K), leading to activation of its downstream effectors, particularly AKT, and promotion of cell migration and invasion [14]. Moreover, with or without the assistance of FAK, SFK could phosphorylate additional substrates, impinging on multiple pro-mitogenic or pro-survival pathways including the Ras-ERK and PI3K-AKT pathways [4]. Additionally, FAK- and SFK-regulated Rac and Cdc42 signaling can activate ARP2/3 complex and LIM kinase to induce actin polymerization, which is necessary for cell migration [15].

Functionally, integrin mediates bidirectional signal crosstalk including “outside-in” signaling where ECM-engaged activation of integrin triggers focal adhesion formation, and “inside-out” signaling where signals inside the cell (e.g., Talin and Kindlin biding) activate the integrin for binding to the extracellular ligands [4, 16]. Through this bidirectional linkage, integrins provide communication between cell and microenvironment, function as a mechano-sensor and force transducer, and coordinate actin cytoskeleton to modulate an array of important biological processes such as cell adhesion, migration, proliferation, differentiation, and apoptosis (Fig. 1b), which are frequently mis-regulated in cancers.

## Roles of integrins in solid cancer

Cancer is a dynamic developmental disorder. Evidence from in vivo genetic studies has established integrins as vital factors regulating development [17]. Consistently, there are several human diseases linked to defects in integrin signaling (e.g., loss of β2 integrin function leads to an autosomal recessive disorder of the immune system called leucocyte adhesion deficiency type 1 (LAD-1) [18]). Given their important roles in diverse development contexts, integrins are expected to play pivotal roles in cancer development and progression (Table 1). For example, mice lacking β3 integrins or both β3 and β5 integrins not only support tumorigenesis, but have enhanced tumor growth as well [19]. Under hostile conditions, tumor cells are generally able to repurpose the multifarious functions of integrins to favor their survival, proliferation and migration (Fig. 1b). Indeed, accumulating evidence has indicated, undeniably, that integrins are involved in almost every step of cancer development, including cancer initiation and proliferation, local invasion and intravasation into vascular system, survival of circulating tumor cells (CTCs) in the blood stream, priming of the metastatic niche, extravasation into the secondary site and metastatic colonization of the new tissue (see [12, 20] for more detail). Importantly, integrin-mediated pathways are also frequently connected to the development of drug resistance [12]. A key property that endows integrin with such pro-tumorigenic or oncogenic functions is that they often regulate stemness, thus eventually facilitating tumor progression.

**Table 1:** Integrin functions in different solid cancer types

Integrin	Ligands/signaling partners	Cancer type	Function	References
β1	Src/AKT	Lung	Promotes chemoresistance against EGFR inhibitor Erlotinib	[35]
α2β1	Collagen type I	Prostate	Promotes prostate cancer cell invasion and skeletal metastasis	[36]
	CDH-17	Colon	Interacts with CDH17 to induce FAK and Ras activation and thus promotes tumor growth and liver metastasis	[37]
α3β1	Rho/Yap	CRPC	Suppresses anchorage-independent growth and metastasis	[38]
α6	AKT/ERK	Breast	Expresses highly in breast cancer vs. normal cells and enhances radiotherapy resistance	[39]
α7	Laminin	Glioblastoma	Correlates negatively with patient survival and promotes growth and invasiveness of CSCs	[40]
α9β1	β-catenin/E-cadherin	Lung	Promotes EMT, tumor growth, vasculogenesis and metastasis	[41]
α10β1	Collagen and Laminin	Glioblastoma	Upregulates in both glioblastoma tissue and cells, correlates with high-grade gliomas, and promotes cell proliferation and migration	[42]
β3	TGFβ	NSCLC	Accelerates cancer cell adhesion to lymphatic endothelium after TGF-β exposure, and combined targeting of β3 integrin and TGFβ reduces lymph node metastasis	[43]
αvβ3	OPN/FAK	NSCLC	Enhances cell proliferation and EGFR-TKI resistance	[44]
	Survivin	CRPC	Promotes anchorage-independent cell growth and enhances IR resistance via stabilization of Survivin	[45]
	KRAS/RalB	Lung/Breast/ Pancreas	Recruits KRAS and RalB to activate TBK1 and NF-κB, leading to enhanced stemness and RTK inhibitor resistance	[46]
αvβ6	MMP9	SCC	Promotes invasion in an MMP9-dependent manner	[31]
	JNK1/Survivin	CRPC	Promotes anchorage-independent growth and cancer progression via activation of androgen receptor	[28]
α6β4	BNIP3L	TNBC	Induces BNIP3L-dependent mitophagy and lactate production in CAFs, which in turn promotes EMT, proliferation and invasion	[47]
β8	ECM	PDAC	Regulates positively cancer cell radiochemoresistance, intracellular vesicle trafficking, and autophagy	[48]

*CRPC* Castration-resistant prostate cancer, *CSCs* cancer stem cells, *EMT* epithelial-mesenchymal transition, *PDAC* Pancreatic ductal adenocarcinoma, *TNBC* triple negative breast cancer, *NSCLC* Non-small-cell lung cancer, *ECM* extracellular matrix, *TKI* tyrosine kinase inhibitor, *RTK* receptor tyrosine kinases, *IR* ionizing radiation, *SCC* squamous-cell carcinoma, *CAFs* cancer-associated fibroblasts

### Pan-cancer mutational landscape of genes encoding integrins

**Fig. 2:**
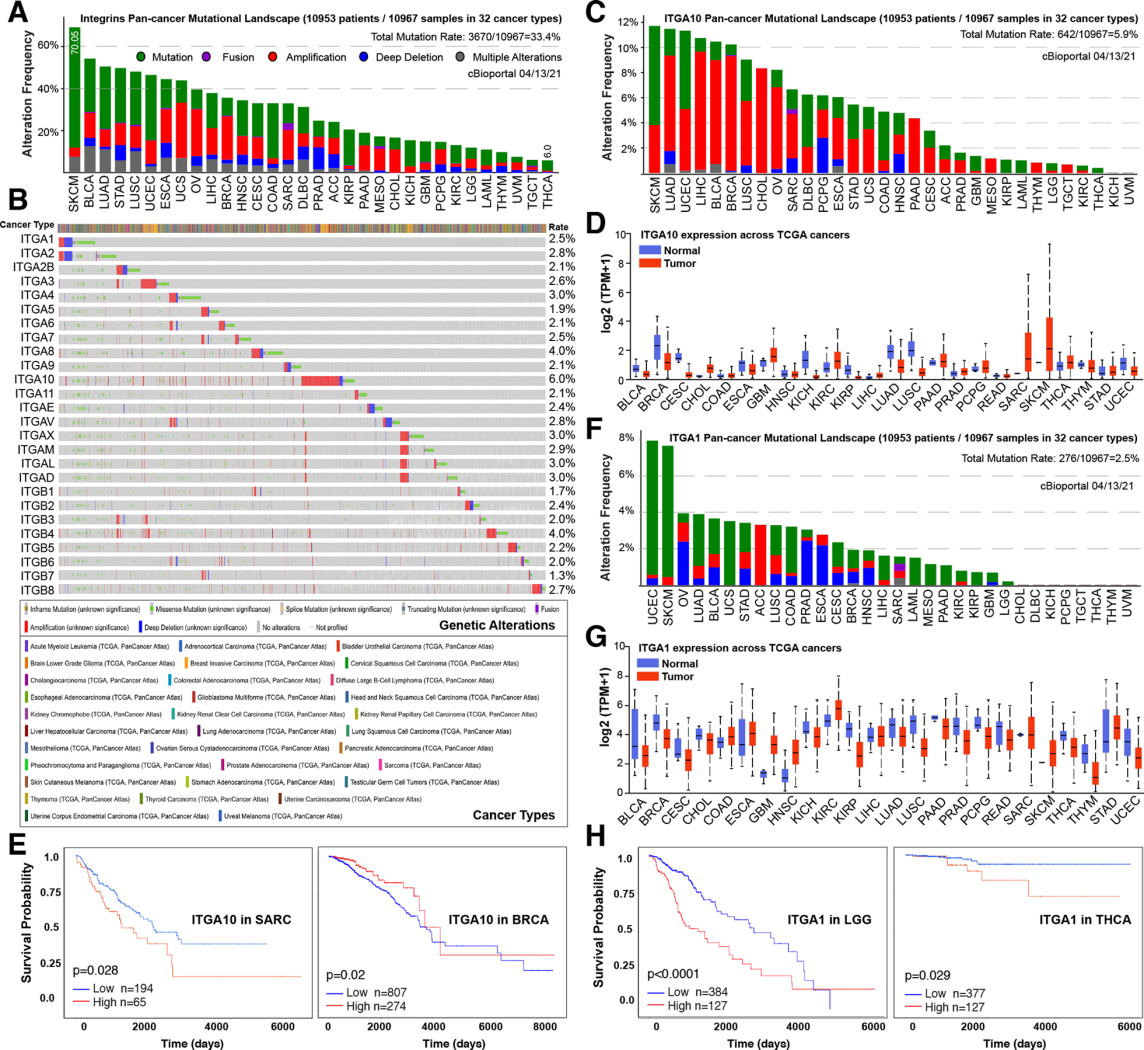
The clinical relevance of integrin genes. **a, b** Mutational landscape of genes encoding integrins in 32 human cancers. Shown are bar plots illustrating the cumulative aberration frequencies of all 26 integrin genes combined (**a**) and heatmaps displaying the genetic alterations of individual integrin gene (**b**) cross human cancer types. **c–e** The clinical relevance of the most amplified integrin subunit *ITGA10*, encoding α10 integrin, in 32 human cancers. Shown are pan-cancer mutational landscape of *ITGA10* (**c**), pairwise comparison of *ITGA10* mRNA expression between normal and tumor tissues in indicated TCGA cancer types (**d**), and Kaplan–Meier plots illustrating *ITGA10* as an unfavorable and a favorable gene associated with patient overall survival in indicated cancer types, respectively (**e**). (**f–h**) The clinical relevance of the most deleted integrin subunit *ITGA1*, encoding α1 integrin, in 32 human cancers. Shown are pan-cancer mutational landscape of *ITGA1* (**f**), pairwise comparison of *ITGA1* mRNA expression between normal and tumor tissues in indicated TCGA cancer types (**g**), and Kaplan–Meier plots illustrating *ITGA1* as an unfavorable gene associated with patient overall survival in indicated cancer types (**h**). Data derived from TCGA pan-cancer analysis encompassing 10,953 patients representing 32 cancer types was viewed through cBioPortal. Gene expression and survival analysis were visualized via online database UALCAN (http://ualcan.path.uab.edu/index.html)

Changes in the ECM and the repertoire of integrins on tumor cells contribute to deregulation of integrin signaling in cancer [4]. Altered integrin expression patterns have been frequently observed in, and also causally linked to, diverse types of cancers [12]. Examples of the roles of integrins played in human cancers are summarized in Table 1. While the majority of investigated integrins are pro-tumorigenic, there are ones suppressing tumor development and/or metastasis. For instance, in breast cancer (BCa), while most β1 integrins, and more specifically α3β1 integrin, are necessary for mammary tumorigenesis [21], α2β1 integrin (a receptor for collagen and other matrix molecules that highly expresses in normal breast epithelium) is a metastasis suppressor (but with little impact on primary tumor growth) in a mouse model of BCa [22]. To systematically uncover the underlying mechanisms of integrin dysregulation, we surveyed the mutational landscape of 26 genes encoding all human integrins across 32 human cancer types offered by current The Cancer Genome Atlas (TCGA) DNA sequencing effort through cBioPortal [23]. Results showed that integrin pathway represents, collectively, a frequently mutated pathway in human cancers with an average rate of 33.4% (Fig. 2a), ranging from 6.0% in thyroid carcinoma (THCA) to 70.05% in skin cutaneous melanoma (SKCM). However, the mutation frequency is very low at individual gene level (average of 3.0%) (Fig. 2b), suggesting a limited contribution of genomic alteration to the transcriptomic dysregulation. Notably, amplification represents one of the main forms of alterations (e.g., in ovarian (OV), uterine (UCS), breast (BRCA) tumors) (Fig. 2a), potentially suggesting that a global upregulation of integrins may be tumorigenic.

To gain a better understanding of the potential roles played by individual integrins in different cancer-contexts, we chose *ITGA10* and *ITGA1* for further analysis in detail. Interestingly, *ITGA10*, encoding the α10 integrin, is the most amplified gene in a number of cancers (Fig. 2c), and consistent with this genomic alteration, it is consequently dysregulated in the majority of cancers (Fig. 2d). Differential gene expression analysis comparing tumor to normal tissues indicated that *ITGA10* is markedly upregulated in sarcoma (SARC), SKCM and cholangiocarcinoma (CHOL), potentially due to amplification (Fig. 2c, d). However, being highly amplified in a small proportion of patients bearing lung adenocarcinoma (LUAD), bladder urothelial carcinoma (BLCA), breast invasive carcinoma (BRCA) and lung squamous cell carcinoma (LUSC), the expression of *ITGA10* is conversely decreased in these tumors at population level (Fig. 2d), suggesting other mechanisms involved in its transcriptional regulation. Regardless of the genomic alterations, a prognostic value of an integrin is usually determined by its expression. Consistent with its upregulation in SARC and downregulation in BRCA, a higher level of *ITGA10* correlated with poor and better patient survival, respectively (Fig. 2e). This highlights that *ITGA10* functions in a cancer-specific manner. Moreover, although genomic alteration is a rare event for *ITGA1* at the population level given an average mutation rate of 2.5%, it is the most frequently deleted integrin gene in human cancers (Fig. 2f). Considering the fact that *ITGA1*, encoding the α1 integrin, is found pervasively downregulated in the majority of human cancers (Fig. 2g), it is conceivable that α1 integrin may play a tumor suppressive role. In support, clinical prognostic analysis unraveled that cancer patients with its higher expression survive longer (Fig. 2h). Contrastively, an upregulation of *ITGA1* is also observed in few numbers of cancer types such as kidney renal clear cell carcinoma (KIRC) and glioblastoma multiforme (GBM) (Fig. 2g), indicating again a context-dependent function for individual integrin genes (Table 1).

### Integrin αvβ6: a pleiotropic and oncogenic factor

**Fig. 3:**
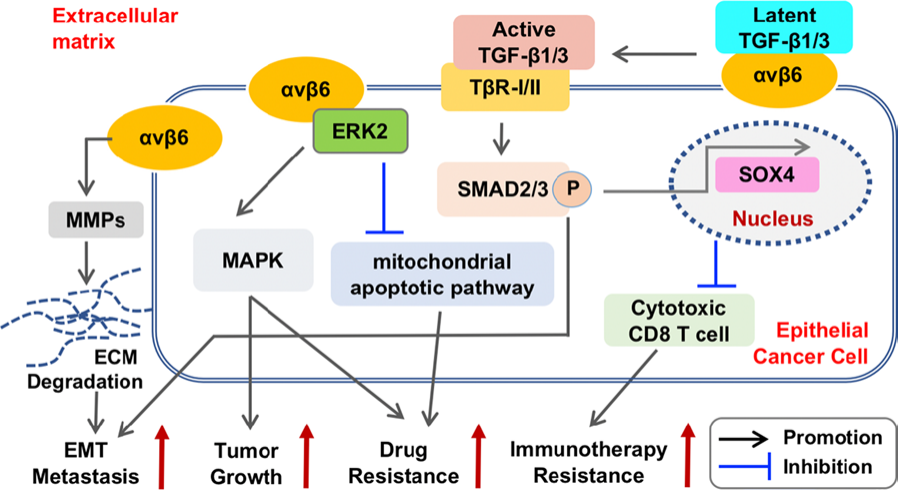
Integrin αvβ6 signalosome in tumor progression. Studies have suggested multiple pro-tumorigenic roles of αvβ6 integrin, involving distinct downstream pathways, in accelerating tumor progression. For example, the αvβ6 integrin can facilitate cancer cell invasiveness and thus metastasis via increasing MMPs secretion and ECM degradation. By interacting with ERK2, αvβ6 activates MAPK signaling to increase cell proliferation and tumor growth, and to inhibit 5-FU-induced cancer cell apoptosis through suppression of the mitochondrial apoptotic pathway (by impairing the cytochrome C release, decreasing the activity of caspase-3 and caspase-9, and upregulating the anti-apoptotic factor BCL-2), leading to drug resistance. Furthermore, the αvβ6 integrin can interact with latent TGFβ and subsequently activate TGFβ-linked pathways to positively contribute to EMT process and metastasis. Alternatively, the activated TGFβ signaling drives expression of cancer stemness factors (e.g., SOX4) to evade immunotherapy. Lines with arrows and perpendicular denote a promotion or an inhibition effect, respectively. P within the circle denotes phosphorylation. See text for details

Among the integrin family, αvβ6 represents one of the most studied integrins so far, in that (1) it is the only heterodimer that can be formed by the β6 subunit [24]; (2) it is barely expressed in healthy adult epithelium, but strongly induced during embryogenesis, tissue repair and tumorigenesis [25]; and (3) it has been found overexpressed in many cancers (e.g., breast [26], gastric [27], prostate [28] and colorectal [29] cancer). Clinically, upregulation of αvβ6 is closely associated with tumor aggressiveness and serves as an independent unfavorable prognostic indicator [29, 30]. Mechanistically, it has been reported that, upon serum or EGF stimulation, αvβ6 binds directly to ERK2 and activates downstream MAPK (as well as other signaling) pathway critical for tumor growth (Fig. 3). Alternatively, integrin facilitates invasiveness through modulating proteolytic activity of ECM via matrix metalloproteinases (MMPs). Induction of αvβ6 in squamous carcinoma (SCC) increases MMP-9 secretion and subsequent ECM degradation, leading to tumor cell survival and metastasis [29, 31]. Moreover, studies have demonstrated that αvβ6 integrin can enhance chemotherapy and radiotherapy resistance. By activating ERK/MAPK signaling and inhibiting mitochondrial apoptotic pathway, αvβ6 protects colon cancer cells from 5-FU induced growth inhibition and apoptosis [32]. Additionally, αvβ6 can also interact with the latent transforming growth factor β (TGFβ) complexes and subsequently releases the active TGFβ from the complex, which then binds to its receptor and activate TGFβ/SMAD pathway [33]. In turn, TGFβ induces epithelial mesenchymal transition (EMT) process that aids metastasis. In triple negative BCa (TNBC), studies indicated that αvβ6 positively regulates expression of SOX4 (a TGFβ target gene) and the SOX4-driven immune evasion pathway [34]. Preclinically, treating tumors with an αvβ6 blocking antibody that inhibits activation of TGFβ and SOX4 expression exhibited reduced invasiveness and, simultaneously, enhanced sensitivity to T-cell mediated immunity [34]. Altogether, these studies establish generally αvβ6 as an oncogenic, but pleiotropic, factor in distinct tumorigenic contexts (Fig. 3) and as an attractive therapeutic target.

## Integrin in control of tumor stemness

Human cancer is a heterogeneous disease with virtually all tumors containing phenotypically and functionally distinct subsets of cells [49]. Cancer stemness, referring to the stem cell (SC)-like phenotype of cancer cells, has been widely recognized as a vital player in different aspects of tumorigenesis [50, 51]. In general, cancer stemness increases along with tumor progression [51]. Among the diverse subsets of cancer cells residing in a tumor, there is a small population of stem-like cancer cells (i.e., cancer stem cells, CSCs) that harbor self-renewing, differentiation and tumor initiating capability. Mounting evidence has established CSCs as drivers of tumor progression, treatment resistance, disease relapse and metastasis [49, 51]. Like normal SCs that are typically associated with a particular local niche, the tumor microenvironment (TME) dictates the fate of CSCs by providing cues to direct their biological behavior. Integrin, as the bridge transmitting “outside-in” and “inside-out” signals (Fig. 1b), is essential for SCs to sense and respond to diverse cues in both normal and diseased tissues [4]. In support, increasing studies have unraveled that integrins could function as both phenotypic markers and functional regulators of CSCs, opening another layer of complexity of CSC regulation, as well as opportunities to devise integrin-targeted therapeutics to impede cancer stemness [50, 52].

### Integrins function as CSC markers

Previously, a number of cell surface markers (e.g., CD44, CD90, CD133) have been extensively characterized to phenotypically mark CSC subpopulations in both cell cultures and clinical samples for many cancer types [51]. Notably, these markers often also mark the normal SCs and lack organ-specificity due to their broad expression repertoire cross tissues, highlighting a need in searching for other markers that can better and more precisely characterize CSCs. In the past decade, another group of transmembrane proteins that have attracted global attention are integrins, owing to their cell surface location and established contributions to tumor evolution [53]. One of the most well-studied integrins reported as SC and CSC markers is α6 (CD49f), a laminin binding receptor. The α6 integrin is highly expressed in embryonic and neural SCs and studies have reported that α6^+^ glioblastoma cells are capable of self-renewal and multi-lineage differentiation, establishing α6 as a CSC marker [54]. Interestingly, compared to CD133 alone which is not CSC-exclusive as CD133^−^ glioblastoma cells also retains tumor initiation ability, a combination of α6 and CD133 better defines glioblastoma CSCs [54]. Notably, besides glioblastoma, an enrichment of α6 is also observed in a variety of CSCs residing in different tumor types (e.g., breast, prostate, colon) [51], suggesting a broader application. For example, integrin α6β3 is commonly used as a marker for luminal progenitors in the mouse mammary gland and in their ErbB2-transformed cancerous derivatives [55]. Recently, integrin α7 was identified as a functional CSC marker in oesophageal squamous cell carcinoma (OSCC) [56]. The α7^+^ cells exhibited an enhanced stemness, including self-renewal, differentiation and chemotherapy resistance. Clinically, a high frequency of α7^+^ cells in OSCC tissues is significantly associated with poor differentiation, lymph node metastasis and worse prognosis. Interestingly, α7 is found co-expressed with CD90 (a previously defined CSC marker in OSCC), but not all CD90^+^ cells are α7^+^. The CD90^+^ α7^+^ double positive cells possessed cardinal CSC properties as CD90^−^ α7^−^ cells barely formed tumors when implanted in vivo, suggesting that α7 can further stratify CD90^+^ population towards stemness compared to CD90^+^ alone. Mechanistically, α7 regulates CSC properties through activation of FAK-mediated signaling pathways [56]. Similarly, there are other integrins that have been suggested to be CSC biomarkers in different solid cancer types (Table 2).

**Table2:** Integrins function as CSC markers in different solid cancers

Integrin subtype	Cancer type	Functions	References
β4	TNBC	Identifies a CSC-enriched population with partial mesenchymal traits	[78]
	PDAC	High level of β4 expression significantly correlates with stemness and EMT	[79]
	Prostate	Sustains the self-renewal of putative CSCs and promotes tumorigenesis by amplifying ErbB2 and c-Met signaling in tumor progenitor cells	[80]
α6	Glioblastoma	Co-expresses with conventional glioblastoma CSC markers and enriched for CSCs	[54]
α7	OSCC	Identifies a CSC-enriched population with elevated expression of SC genes and EMT features. The α7 co-expresses with the traditional CSC marker CD90 but further stratifies and marks a more tumorigenic subset	[56]
β3	Breast	A luminal epithelial progenitor marker that identifies a CSC population in mouse models of mammary tumorigenesis	[81]
β8	Glioblastoma	Overexpresses in and maintains glioblastoma CSC, and its overexpression induces radio-resistance and is correlated with poor prognosis	[82]
αvβ3	Breast	Regulates adult mammary SCs during pregnancy, and activates Slug in BCa cells to increase CSC features such as tumorsphere formation and tumor initiation	[83]
α6 and β3	Breast	α6^high^β3^high^ identifies a CSC-enriched population with enhanced tumorsphere formation and drug resistance to pacitaxel and doxorubicin in mouse Her2/neu transgenic breast tumors	[55]
α2β1	NSCLC	Exosomes derived from NSCLC cells carrying low levels of miR-34c-3p induce upregulation of α2β1, which promotes cancer cell invasion and migration	[84]
	Colon	Enhances metastatic capability and stemness of colorectal cancer cells via PI3K/AKT/Snail axis	[85]
β1	HNSCC	Promotes stemness, chemoresistance and tumor-forming capacity of cancer cells	[86]
	OSCC	Overexpresses in stem-like cancer cells and enhances cell proliferation, migration and tumorsphere formation	[87]

*CSC* cancer stem cells, *TNBC* triple-negative breast cancer, *PDAC* pancreatic ductal adenocarcinoma, *OSCC* oral squamous cell carcinoma, *NSCLC* Non-small-cell lung cancer, *HNSCC* head and neck squamous cell carcinoma, *EMT* epithelial-mesenchymal transition

### Integrins regulate CSCs in tumor development and progression

In addition to serve as CSC markers, there is overwhelming evidence that integrins and integrin signalosome play crucial roles in potentiating CSCs function [2, 51], and CSCs usually coopt niche-integrin signals to fuel their expansion [4]. Here, by providing cases in point, we highlight the integrin β1, α6 and αv, owing to the fact that they are the most studied ones in regulation of CSCs. Many other integrins that functionally regulate CSCs are also briefly described in Table 2.

#### Integrin β1

The β1 subunit (CD29) can form dimers with the biggest pool of at least 10 different α chain partners (α1-9 and αν). It thus has a central role in engaging the TME owing to its ability to bind a broad spectrum of ECM and cell adhesion molecules. In support, studies have reported that an increased level of β1 integrin correlates with tumor progression, metastasis and therapy resistance in many cancer types [57]. Molecularly, multiple integrin-dependent and cancer-related pathways (such as FAK, ERK/MAPK, Src, AKT and Ras) are activated as a result of increased β1 level, empowering promotion of tumor growth and treatment resistance [4]. Interestingly, it has been reported that squamous cell carcinoma (SCC) contains two highly tumorigenic CSC populations that differ in CD34 levels but are enriched for integrins and coexist at the SCC–stroma interface [58]. CD34 was previously identified as a hair follicle SC marker that can be used to purify a skin CSC-like population [51]. Specifically, the tumor initiation ability of SCC CSCs measured by serial limit-dilution transplantation assays seems to be governed by α6β1 but not CD34, as only α6^hi^β1^hi^ populations, regardless of CD34 expression level, can initiate secondary tumors. This suggested that high level of β1 integrin is a better defining marker for tumor initiating CSCs in SCCs [58]. Intriguingly, molecular regulation of the interchangeable states between α6^hi^β1^hi^CD34^hi^ and α6^hi^β1^hi^CD34^low^ is distinct. In one state, activated β1 via binding to its ligand fibronectin (FN1) promotes self-renewal of both CD34^hi^ and CD34^low^ CSC populations; whereas in another state, active TGFβ/TβRII signaling primarily impacted on α6^hi^β1^hi^CD34^hi^ cells to restrain their stemness and induce differentiation. Unsurprisingly, FAK signaling is important for the tumorigenic properties of both CSC subsets [58]. CSC phenotype is tightly linked to metastasis. There is also evidence indicating a critical role of β1 integrin in metastasis, but discrepancy exists. On the one hand, the β1-dependent adhesion was experimentally essential for cancer cells to contact with the subendothelial matrix and thus β1 integrin was necessary for invading through the basement membrane after tumor cells clearing the endothelial wall [12]. Therapeutically, β1 inhibition significantly reduces the formation of metastatic foci in several solid tumor types [12]. On the other hand, depletion of β1 integrin in TNBC epithelial cells reduced tumor growth, but markedly enhanced lung metastasis [59], suggesting a context-dependent effect of β1-targeting therapies.

Besides the cell autonomous activity, integrins can also function in an endocrine manner. Integrin can help cancer cells to build a new beneficial niche by secreting integrin ligands. BCa cells produce tenascin C, a ligand of β1 and β3 integrins, to promote self-renewal of CSCs and to potentiate metastasis-initiating ability. Furthermore, integrin β1 has been proposed to promote resistance to antiangiogenic therapy through elevation of multiple malignant programs facilitated by interactions with the TME [57]. One such program is the vasculogenic mimicry (VM) formation by cancer cells, in which β1 is a critical regulator [60]. Consequently, blocking β1 with OS2966, a neutralizing β1 monoclonal antibody, attenuates aggressive tumor phenotypes in vitro and inhibits growth of antiangiogenic therapy-resistant tumor xenografts in vivo [57], constituting a potential therapeutic opportunity.

#### Integrin α6

As a laminin binding integrin, α6 (CD49f) mainly heterodimerizes with two β chains (β1 or β4) to primarily anchor epithelial cells to laminin in the basement membrane [4]. CD49f has been widely used to mark, and is functionally essential for the maintenance of, CSCs in a number of solid cancer types [51]. Genetic depletion of *ITGA6*, encoding α6 subunit, suppresses CSCs and their tumor initiation capacity in glioblastoma [61]. Mechanistically, α6 positively regulates the expression of fibroblast growth factor receptor 1 (FGFR1) through a ZEB1/YAP transcription complex, leading to increased expression of multiple SC factors in cancer cells [61]. In TNBC, CSCs contain both epithelial and mesenchymal subsets and there are two distinct cytoplasmic domains of the α6 integrin (α6A and α6B) generated by alternative splicing. These two splicing variants drive, interestingly, opposite phenotype: α6Aβ1 maintains an epithelial phenotype; whereas α6Bβ1 defines the mesenchymal population and is necessary for CSC function [62]. But in both phenotypes, α6 upregulates expression of a key SC factor, BMI-1, via activation of FAK signaling to contribute to CSCs [63]. In prostate cancer (PCa), miR-25 functions as a tumor suppressor in highly metastatic prostate CSCs defined by ALDH^hi^ phenotype. One of the key targets of miR-25 is α6. Experimentally, overexpression of miR-25 significantly reduces the invasive behavior of PCa cells in vitro and blocks extravasation and hence metastatic colonization in a zebrafish model in vivo [64].

#### Integrin αv

The αv integrin forms heterodimers with one of the five different β subunits (β1, 3, 5, 6 or 8) and the αv-containing integrins recognize RGD peptide motifs in various ECM ligands [2, 12]. Studies have linked an aberrant regulation of αv integrins, particularly αvβ3 and αvβ5, with poor patient outcome and higher incidence of metastasis for many epithelial cancers [4, 65]. Functionally, expression of the αv integrin is reported to be tightly associated with a CSC phenotype, because it is unique in its ability to cluster on the surface of non-adherent tumor cells where it contributes to anchorage-independent growth [4, 66]. It is well established that anchorage-independent growth is a hallmark of tumor stemness and progression. In support, αvβ3 was found necessarily and sufficiently to reprogram tumor cells toward a CSC phenotype (including tumor initiation, self-renewal and, particularly, resistance to receptor tyrosine kinase (RTK) inhibitors such as erlotinib) in breast, lung and pancreatic carcinomas [46]. To do so, αvβ3 interacts with Galectin-3 independent of its ligand binding domain and then recruits KRAS and RalB to the plasma membrane, leading to activation of NF-kB signaling via TBK1 [46]. Similarly, in gastric cancer, αvβ3 mediates intercellular adhesion in multi-cellular aggregates (MCAs) and activates GLI1 through a non-classic ERK1/2 pathway and the classic Hedgehog pathway to maintain CSCs. Importantly, the MCAs were found responsible for peritoneal metastasis [67]. Consequently, αvβ3 integrin is now established as a CSC marker in multiple solid tumor types [46]. Similarly, αvβ5 has also been reported to promote stemness-associated invasion and metastasis of tumors growing in preirradiated stroma [68] and brain metastases of lung cancer [69]. The αvβ5 is recently identified as a CSC marker essential for glioblastoma maintenance and Zika virus (ZIKV) infection [70]. As a neurotropic virus, ZIKV has potentially an oncolytic activity against glioblastoma CSCs characterized by αvβ5 and SOX2 expression. Therapeutic inhibition of αvβ5 by blocking antibodies attenuated ZIKV infection [70]. Furthermore, EMT accelerates stemness and integrin signaling exerts its most evident effect during EMT [4]. The β8 heterodimerizes exclusively with the αv to form αvβ8 integrin, which primarily binds to RGD sequences in ECM-bound latent TGFβ1 and TGFβ3 (inactive) protein ligands and mediates subsequent ligand activation and functional TGFβ signaling. Data mining in TCGA database suggested that β8 expression in glioblastoma samples is correlated positively and negatively with the expression of numerous SC/EMT and neural differentiation markers, respectively [71]. Consistently, αvβ8 integrin is highly expressed in glioblastoma CSCs and essential for their self-renewal and lineage commitment during tumorigenesis. Molecularly, αvβ8 promotes tumor development, in part, by driving TGFβ1-induced DNA replication and CDK1- and CDKN1A/p21^cip^-mediated cell cycle progression [72].

All together, these findings reflect that integrins are implicated in nearly every step of cancer progression from primary tumor development to treatment-resistance to metastasis [12]. Strikingly, and in line with the idea that tumor stemness is the main driver of cancer evolution, many integrin-derived signals are frequently reported to be important for CSC functions. Therefore, the integrin control of stemness represents another therapeutic strategy of targeting specific integrins to suppress CSCs via modulation of cell adhesion and integrin/ECM interactions [4, 12, 16]. However, it is worth noting that studies have also shown that integrins can regulate tumor stemness independent of their ECM interaction [4]. Interestingly, this adhesion-independent functions of integrins trigger pathways distinct from the typical signaling cascade and cytoskeletal links (Fig. 1b). In such cases, it is conceivable that integrin antagonists that compete for ligand binding or impair ligand binding ability would fail to produce desirable therapeutic benefits (see below), thus warranting the development of innovative strategies.

### Integrins in cancer-derived exosomes aid metastasis

Metastasis is the leading cause of cancer mortality. The disseminated cancer cells must adapt to colonize and thrive at the “foreign” metastatic site. In recent years, exosomes have been proposed as the “vanguard” to prime distant organs to form pre-metastatic niche [73]. Exosomes are small membrane vesicles (30–100 nm in diameter) secreted by cancer cells [74]. By capsuling functional biomolecules (e.g., proteins, lipids, RNA and DNA), exosomes are both short- and long-distance mediators of intercellular communication through modulation of the recipient cells on ligand–receptor interaction and/or cargo release [74]. Integrins are the most abundantly expressed receptors on the surface of exosomes, and given their ECM modulating abilities, exosome-carrying integrins have been recognized to actively participate in metastasis [73]. In PCa, tumor cells secreted αvβ6 which is not detectable in normal human prostate, and transferred it intercellularly via exosomes to an αvβ6-negative recipient cell, finally enhancing cell migration and metastasis in a paracrine fashion [75]. The castration resistant PCa (CRPC) is currently a lethal variant of aggressive PCa [49]. Emerging evidence indicated that small extracellular vesicles (sEVs) secreted by αvβ3-expressing CRPC cells are capable of reprogramming adenocarcinoma cells towards the most aggressive neuroendocrine phenotype, whereas αvβ3-nonexpressing sEVs have minimal impact on tumor growth [76]. In pancreatic cancer, an array of studies have also identified roles of the exosomal integrins (e.g., β4, α6β4, αvβ5) in disease progression and metastasis [73]. Broadly, a comprehensive proteomic survey has portrayed the diverse integrin repertoires in exosomes derived from distinct types of primary tumors [77], indicative a selectivity of exosome cargo packaging. Functionally, these distinct integrin heterodimers determine organotropic metastasis by interacting with resident cells at targeted destination [77]. For instance, exosomal α6β4 and α6β1 integrins bind to pro-inflammatory S100A4^+^ fibroblasts and SPC^+^ epithelial cells and help target metastatic cells to the lung; whereas αvβ5 specifically bind to F4/80^+^ macrophages and is linked to liver metastasis [77]. Collectively, these findings unravel another layer of complexity in integrin-mediated control of tumor progression, especially metastasis.

## Integrins as therapeutic targets in cancer

Integrins modulate mainly cell–cell and cell–ECM interaction, and in certain contexts activate encountered growth factor receptors (GFRs), to amplify the signaling cascade to ultimately alter cell behavior. An association of integrins with specific GFRs can result in their partial activation: αvβ3 can partner with the receptors of insulin, platelet derived growth factor (PDGF), and vascular endothelial growth factor (VEGF); whereas α5β1 often associates with the EGF receptor (EGFR) [4, 9]. Moreover, α5β1 can also binds to fibronectin to induce ligand-independent activation of the RTK MET; whereas α6β4 integrin regulates MET oncogenic signaling [12]. Obviously, because of the functional importance and their ligand binding and regulatory sites are extracellular (which render accessibility to diverse therapeutic interventions), integrins are thought idea drug targets [12]. Notably, intensive pre-clinical studies have linked an altered expression or functionality of integrins to human tumorigenesis, directly leading to a focus on development of agents targeting integrins over the past decades [88]. In the non-cancer treatment field, there are successful anti-integrin drugs on the market. The β3 blocking antibody, Abciximab, has been used to prevent clot formation during high-risk coronary angioplasty [89]. Natalizumab, a pan-α4 inhibitory antibody, is now wildly used to treat multiple sclerosis (MS) and Crohn’s disease. It is uniquely efficacious in MS, although a side-effect of developing fetal progressive multifocal leukoencephalopathy was observed [90]. Natalizumab has been proven by the US Food and Drug Administration to treat patients under rigorous monitoring for John Cunningham polyoma virus [90]. In the setting of cancer management, although translating the basic research findings into clinic is challenging, efforts are been made towards development of effective integrin-blocking therapeutics for treating deadly cancers.

### Targeting integrins by monoclonal antibodies

Five out of the nine integrin-targeting drugs made to the clinical trial stages are anti-integrin αv (Table 3). Notably, the αv subunit is usually not expressed in epithelial cells and is hence largely nonessential for development (justifying αv as a desirable target), but has been implicated in tumor angiogenesis and metastasis [16]. Experimental blocking of αv-containing integrins’ ligand binding function inhibits endothelial cell-mediated angiogenesis, accompanied with reduced tumor cell proliferation, migration, and metastasis [16]. These findings greatly stimulate the efforts of developing αv antagonists for clinical use. Intetumumab (CNTO 95), a fully human monoclonal antibody, recognizes the αv integrins with demonstrated blocking activity against αvβ3 and αvβ5 [91]. In BCa, intetumumab treatment interrupts integrin αv-activated pathways associated with focal adhesions and cell motility, thus leading to reduced tumor growth and lung metastasis in mouse models [91]. In a phase I clinical trial, CNTO 95 was safely tolerated in patients with malignant solid tumors [92]. However, results from a phase II trial in melanoma demonstrated no statistically significant efficacy for, although there seemed a trend of, an improved overall survival with CNTO 95 treatment alone or combined with dacarbazine (a chemotherapy) [93].

**Table 3:** Examples of clinical trials evaluating integrin targeting drugs

Drug	Target and mechanism	Therapy strategy	NCT#	Clinical phase	Cancer type	Status and Refs
Intetumumab (CNTO 95)	Pan αv-integrin antibody	Alone or in combination with Dacarbazine	NCT00246012	Phase I/II	Stage 4 melanoma	A nonsignificant trend towards improved OS was observed [93]
		In combination with docetaxel and prednisone	NCT00537381	Phase II	Metastatic CRPC	All efficacy end-points favored placebo but not intetumumab, including PFS, tumor response, PSA response, suggesting no beneficial effects [121]
Abituzumab (EMD 525797)	Pan αv-integrin antibody	In combination with LHRH agonist/antagonist	NCT01360840	Phase II	Asymptomatic or mildly symptomatic metastatic CRPC	Favorable safety profile and specific activity in PCa–associated bone lesions were observed, but PFS was not significantly extended [94]
		In combination with cetuximab and FOLFIRI	NCT03688230	Phase II	KRAS wild-type metastatic CRC with high ανβ6 expression	Withdraw (the primary PFS end point was not met) [95]
Etaracizumab (MEDI-522 or Vitaxin)	αvβ3 inhibiting antibody	In combination with dacarbazine	NCT00066196	Phase II	Metastatic melanoma	Terminated due to no clinically meaningful improvement over dacarbazine alone was observed [98]
Volociximab	α5β1 inhibiting antibody	Monotherapy	NCT00516841	Phase II	Platinum-resistant advanced epithelial ovarian or primary peritoneal cancer	Therapy was well-tolerated, but terminated due to lack of efficacy [100]
*Cilengitide	RGD peptide mimetic inhibiting αvβ3 and αvβ5	In combination with Cisplatin, 5-FU, and Cetuximab (PFE combo)	NCT00705016	Phase I/II	Metastatic HNSCC	Neither of the cilengitide-containing regimens demonstrated a PFS benefit over PFE alone [122]
		In combination with temozolomide (standard chemoradiotherapy)	NCT00689221	Phase III	Glioblastoma with methylated MGMT promoter	Failed due to no improvement in outcomes, although no additional toxicity was observed [104]
*ATN-161	A noncompetitive small peptide inhibitor of β subunits	Monotherapy		Phase I	Advanced solid tumors	Drug was well tolerated but no objective responses were observed [105]
		Monotherapy	NCT00131651	Phase II	Advanced renal cell cancer	Terminated without published clinical results
*E-7820	Reducing α2 integrin expression	In combination with Cetuximab	NCT00309179	Phase II	Metastatic and refractory CRC	Therapy was well-tolerated, but terminated due to lack of efficacy [107]
*GLPG0187	A broad-spectrum integrin receptor antagonist	Monotherapy	NCT01313598	Phase I	High-grade glioma and other advanced solid tumors	Drug was well tolerated with a dose-proportional pharmacokinetics profile, but failed to show signs of efficacy [108]
*MK0429	Non-peptide small molecule inhibitor of αvβ3 integrin	Monotherapy	NCT00302471	Phase I	CRPC with bone metastases	Drug was well tolerated and showed early reduction of bone turnover, although PSA was unexpectedly increased during the treatment [109]

*LHRH* luteinizing hormone-releasing hormone, *CRPC* castration-resistant prostate cancer, *PSA* prostate specific antigen, *PCa* prostate cancer, *PFS* progression-free survival, *OS* overall survival, *HNSCC* squamous cell carcinoma of the head and neck, *CRC* colorectal cancer

*Small molecule integrin inhibitor

Abituzumab (EMD 525797), a fully human de-immunized monoclonal antibody that targets all five αv integrins, is de-glycosylated and expected to not cause antibody dependent cellular cytotoxicity (ADCC) [2]. In a large phase II trial in CRPC with 60 patients in each arm, abituzumab was evaluated in combination with hormone agonist/antagonist therapy in chemotherapy naïve patients with progressive bone lesions. Results showed that although the incidence of bone lesion progression was decreased, but the extended progression free survival (PFS) was not improved between the treated and placebo groups [94], suggesting a limited clinical efficacy. Abituzumab has also been examined in metastatic colorectal cancer (mCRC) in a phase I/II trial. A favorable safety profile was observed in combination with standard of care (EGFR inhibitor cetuximab plus chemotherapy irinotecan) for mCRC, but the primary end point (i.e., PFS) was not met [95]. A biomarker analysis investigating the correlation between integrin expression and treatment outcome suggested that some mCRC patients with a high αvβ6 expression did benefit from abituzumab-based therapy [95], indicating a need of up-front stratification based on αvβ6 levels for better clinical practice.

LM609, a functional blocking monoclonal antibody of integrin αvβ3, has demonstrated anti-angiogenic activity in preclinical animal studies [96]. In a phase I trial, etaracizumab, a humanized version of LM609 (also named Vitaxin commercially) directly against conformational epitope of αvβ3 integrin, exhibited anti-tumor activity, but seemingly in a manner independent of its antiangiogenic effects, in human melanoma [97]. In a randomized multicenter phase II study of etaracizumab in patients with stage IV melanoma, etaracizumab, as a single agent or combined with dacarbazine (a chemotherapy), displayed acceptable safety profile but did not prolong the survival comparing to dacarbazine alone [98].

Besides the αv integrins, therapeutic targeting of β1 integrin has also shown promising efficacy in reducing tumor burden in pre-clinical models. Volociximab, an α5β1-inhibiting antibody, is reported to block angiogenesis and hence tumor growth in multiple xenograft models [99], but its further development was silenced due to a lack of efficacy in a phase II trial for human solid tumors [100, 101]. A brief description of abovementioned integrin-blocking antibodies that have reached late-stage clinic trials are summarized in Table 3. More information can be obtained in recently published reviews [4, 12, 16].

### Targeting integrins by small molecule inhibitors

The antibody-based therapies have been, unfortunately and largely, disappointing so far. Although there are few small molecule integrin inhibitors that have reached clinical trial stages, considering the distinct intrinsic properties associated with small molecule versus (vs.) antibody in many aspects, small molecule inhibitors (including short peptides) may offer a new avenue to target integrins. The RGD peptide mimetic, cilengitide, that specifically inhibits both αvβ3 and αvβ5 integrins, is the first anti-integrin drug in cancer that has reached the level of phase III trial. Cilengitide has been tested in PCa, SCC of the head and neck and non-small cell lung carcinoma (NSCLC) in phase II trials and glioblastoma (a highly vascularized tumor type) in a phase III trial. It is worth noting that αvβ3 expresses highly on angiogenic blood vessels and their ligand vitronectin is reciprocally and abundantly expressed in high grade glioblastoma. Although in preclinical studies cilengitide effectively impaired the angiogenesis and tumor growth [102], it failed to show a desirable anti-tumor efficacy in patients with recurrent glioblastoma in a phase II trial [103]. Moreover, in a phase III trial, cilengitide was assessed in combination with chemoradiotherapy (temozolomide) in patients bearing newly diagnosed glioblastoma with methylated O-6-methylguanine-DNA methyltransferase (MGMT) promoter (a prognostic biomarker for glioblastoma). Unfortunately, no clear positive outcome was observed and thus cilengitide was discontinued for further development [104]. Another antiangiogenic small peptide ATN-161 (a 5aa peptide Ac-PHSCN-NH2 derived from the synergy region of fibronectin) has also been previously evaluated in a phase I trial in patients with solid tumors [105]. ATN-161 binds exclusively to integrin β chains and inhibits the function of several integrins implicated in tumor angiogenesis and metastasis. Although it was well tolerated at all dose levels, no objective responses were observed (Table 3) [105].

E-7820 is a sulphonamide-based small molecule. It was used to suppress α2 integrin and thus angiogenesis and solid tumor growth in a panel of xenograft models (e.g., colon, breast, pancreas, and kidney) [106]. Unfortunately, E-7820 showed little efficacy towards advanced CRC in a phase II trial combined with cetuximab (an EGFR targeted agent) [107]. Additionally, early phase I trials can be found for another two small chemical inhibitors, GLPG0187 (a broad spectrum integrin receptor antagonist) [108] and MK0429 (an orally active αvβ3 integrin inhibitor) [109]. Both drugs were well tolerated, but the therapeutic efficacy was dismal (Table 3).

Furthermore, several natural product compounds are reported to exhibit promising effects in modulating integrin signaling and are thus under investigation towards clinical use. For example, the water extract of *Gleditsia sinensis* thorns (GST, a traditional medicine used for carbuncles and skin diseases) can suppress the expression of α2 integrin and attenuates migration and adhesion of PCa cells to collagen [110]. D-pinitol, a 3-methoxy analogue of d-chiro-inositol previously identified as an active principle in soy foods and legumes, can reduce the cell surface expression of integrin αvβ3 through reducing FAK phosphorylation, c-Src kinase activity and NF-kB activation, which in turn negatively regulates PCa metastasis [111]. These results establish D-pinitol as a novel anti-metastasis agent worthy further exploration. Curcumin, a bioactive lipophilic polyphenol extracted from the rhizome *Curcuma longa*, has been demonstrated to have a wide pharmacological effects (such as anti-inflammatory, anti-oxidation and anti-tumor activities) [112]. Inspired by its anti-cancer effect against a spectrum of cancer cells accompanied with limited toxicity, curcumin has entered the clinical trial phase and is currently tested either alone or in combination with other drugs in multiple cancer types [113]. Mechanistically, curcumin was reported to regulate distinct integrins in different cancer types. It blocks BCa cell motility and invasion by directly disrupting the α6β4 function, leading to reduction of Akt and ENPP2 (a migration promoting factor) activity. In addition, curcumin also attenuates RCP (Rab coupling protein)-induced ovarian cancer cell invasion by blocking stabilization of β1 integrin and consequently inhibiting FAK and EGFR activation [114]. Ouabain, a plant-derived cardioactive glycoside from the seeds of *Strophanthus gratus* and also recognized as a hormone inhibiting Na^+^/K^+^-ATPase, has been shown to decrease the expression of multiple integrins (α4, α5, αv, and β3, but not β1 and β4) in human lung cancer cells when treated with non-toxic concentrations, leading to suppression of migration [16]. In addition, there are other compounds currently under investigation [16]. Collectively, some of these natural products have yield promising results in preclinical studies, warranting further therapeutic exploration.

### Integrins as probes for cancer imaging and drug delivery

In addition to a usage as drugs, integrin antagonists with high safety can be repurposed to be integrin tracers in cancer imaging and drug delivery [12]. Different radionuclides labeled RGD peptide antagonists of αv-containing integrins have been developed to provide a non-invasive quantitative assessment of certain integrin expression with positron emission tomography (PET, using positron emitting radionuclides such as ^18^F or ^68^Ga) or with single photon emission computed tomography (SPECT, using gamma emitters like ^99m^Tc) scanning [115, 116]. This RGD-based optical imaging probes have been used for patient stratification for antiangiogenic therapies, as well as for monitoring treatment response. For example, 18F-fluciclatide is an RGD sequence based cyclic tripeptide and binds to both endothelial-specific αvβ3 and αvβ5 with high affinity. This compound has shown successful tumor-recognizing ability in various solid tumor models, and has thus been invested as a radiotracer for imaging of tumor angiogenesis in multiple clinic trails [116]. In a recent trial, ^18^F-fluciclatide PET imaging was tested to assess antiangiogenic effect of pazopanib (an RTK inhibitor) in patients with platinum-resistant/refractory ovarian cancer. Administration of pazopanib resulted in a reduction in ^18^F-fluciclatide baseline uptake, predictive of a good clinical outcome [117]. Similarly, ^68^Ga-NOTA-RGD PET is an RGD containing cyclic peptide c (RGDyK) coupled with SCN-Bz-NOTA and has been evaluated in six patients with hepatic metastases of CRC before antiangiogenic combination therapy with FOLFOX and bevacizumab (VEGF inhibitor). Half of the patients with elevated uptake of ^68^Ga-NOTA-RGD showed a partial response to the treatment; whereas the other half had stable or progressive disease [116, 118].

Alternatively, besides applications in monitoring both prognosis and treatment efficacy, the tumor-homing properties of RGD peptides can also be utilized to deliver therapeutically active compounds. Studies have shown that integrin αvβ3-targeted nanoparticles, through biding to RGD, selectively delivered doxorubicin to tumor vasculature and resulted in a 15-fold improvement in anti-tumor and anti-metastatic activity of doxorubicin, while largely eliminating the systemic toxicity such as weight loss associated with doxorubicin [119]. Recently, a report using nanoparticulate delivery of short interfering RNAs (siRNA) targeting β1 and αv integrin subunits has demonstrated the effectiveness of this strategy in retarding tumor progression in a hepatocellular carcinoma (HCC) mouse model in vivo [120].

## Conclusions and perspectives

Integrin and integrin signalosome are implicated in every step of tumorigenesis from primary tumor development to metastasis [12]. Among these implications, cancer stemness and drug resistance are cordially triggered by alterations in integrin expression and function. Various mechanisms deregulate integrin signaling in cancer [4]. Although multiple clinical trials of anti-integrins in several solid cancers have so far yielded disappointing results which cause a discontinuation in these drugs, this therapeutic barrier of current regimens pose both opportunities as well as needs for developing better integrin blockers [66]. A number of reasons are proposed to be underlying the failure of integrin-inhibiting blockers in clinical practice [4, 66]. First, xenograft models are often used in pre-clinical studies, and it is now well-accepted that translatability of such cell-line-derived tumor models to actual situations in clinic is limited. This could be one of the major contributing factors to the lack of reproducibility of preclinical findings. Second, the functional redundancy between integrins, exemplified by the fact that one α integrin can partner with multiple β subunits and different integrin dimers might possess similar functions, creates difficulty in inhibiting integrin-induced adhesion and signaling with a single agent [4]. Also, this could elicit certain side-effects, although integrin-targeting therapies are generally well-tolerated [88]. Third, and notably, current therapeutic strategies aim predominantly to interfere with integrin-ligand interactions, but an oncogenic integrin-downstream signaling can still occur in a ligand-independent manner. In such scenario, targeting both integrin-mediated adhesion and its downstream signaling might be a better approach. Forth, many clinical trials are executed on a mix population and thus it is difficult to pinpoint why certain number of patients response well while the others not. Stratification of cancer patients based on the predictive biomarkers represents a new and practical direction towards a better use of existing drugs.

Thinking differently, there is an emerging and attractive concept of targeting aggressive cancer via combination of integrin-targeted therapy with immunotherapy. Although evidence from clinical trials currently lacks, lines of preclinical studies have established the validity of this novel approach. As a proof-of-concept, combining PD-L1 based immunotherapy with integrin αvβ3-targeted radionuclide therapy (TRT, ^177^Lu-EB-RGD) significantly improved the anti-tumor efficacy and overall survival compared with either treatment alone in a murine MC38 colon cancer model [123]. Besides the traditional antibody- or small molecule-based strategies, integrin itself presents as an effective target for cancer immunotherapies. For example, integrin β4 is recently suggested to be an immuno-target in mouse models of mammary and head and neck tumors [124]. Using ITGB4 protein-pulsed dendritic cells (ITGB4-DC) for vaccination or adoptive transfer of anti-CD3/anti-ITGB4 bispecific antibody (ITGB4 BiAb)–armed tumor-draining lymph node T cells, both immunologic strategies significantly inhibited local tumor growth and metastases in both solid tumor models via preferential killing of ALDH^high^ CSCs over non-CSCs. Interestingly, the therapeutic efficacy of both of these ITGB4-targeted immunotherapies was significantly enhanced by the co-administration of anti–PD-L1 without obvious systemic toxicity [124]. Alternatively, studies have explored the possibility of RGD-binding integrins as targets for antibody Fc effector functions in the context of cancer immunotherapy. Using an integrin-binding peptide fused to the antibody Fc-domain (2.5F-Fc), combined with an engineered mouse serum albumin/IL-2 fusion, various types of tumor mouse models were treated and an improved survival was observed [125]. Specifically, this treatment strategy accelerated the activation of CD8^+^ T cells and natural killer cells by boosting the host immune system, rather than blocking the integrin function, to achieve therapeutic effects. Addition of anti-PD1 therapy to this combination further improved therapeutic responses and predominantly resulted in cures [125]. The chimeric antigen receptors (CAR)-engineered T cells represent a unique and promising cancer immunotherapy [51]. By utilizing a multiple myeloma (MM)-specific mAb (MMG49) specifically recognizing the activated conformation of integrin β7, CAR T cells transduced with MMG49 recognize and preferentially kill MM cells without damaging normal hematopoietic cells in vivo [126]. Considering the upregulation and constitutive activation of many integrins in cancers, the active conformer of certain integrins may present as actionable immunotherapeutic targets.

Although the successful clinical trials based on current methodologies are regrettably few in number, a continued drive to better understand the roles of integrins in tumorigenesis could lead to development of more innovative targeting approaches and a revival in the field [4, 12]. Elimination of CSCs has been a focus, although challenging, in cancer treatment field. Given that integrin repertoire phenotypically identifies, and functionally operates in, CSCs across multiple tumor types, further delineation of the core mechanisms by which integrin signalosome maintains a CSC phenotype could facilitate designing better drugs and developing multi-modal therapies that integrate both conventional or immunological and CSC therapeutic approaches.

## Data Availability

The material supporting the conclusion of this review has been included within the article.

## Abbreviations

ECM: Extra cellular matrix
CSC: Cancer stem cell
TM: Trans-membrane
RGD: Arg-Gly-Asp
VCAM-1: Vascular cell adhesion molecule-1
ICAM-1: Intercellular adhesion molecule-1
MIDAS: Metal ion-dependent adhesion site
PSI domain: Plexin sempahorin integrin domain
EGF: Epidermal growth factor
TMD: Trans-membrane helical domain
PTB domain: Phosphotyrosine binding domain
FAK: Focal adhesion kinase
SFK: Src family kinase
GRB2: Growth factor receptor bound protein 2
ERK: Extracellular signal regulated kinase
MAPK: Mitogen activated protein kinase
PI3K: Pphosphoinositide 3-OH kinase
LAD-1: Leucocyte adhesion deficiency type 1
CTC: Circulating tumor cell
BCa: Breast cancer
TCGA: The cancer genome atlas
THCA: Thyroid carcinoma
SKCM: Skin cutaneous melanoma
OV: Ovarian serous cystadenocarcinoma
UCS: Uterine carcinosarcoma
BRCA: Breast invasive carcinoma
SARC: Sarcoma
CHOL: Cholangiocarcinoma
LUAD: Lung adenocarcinoma
BLCA: Bladder urothelial carcinoma
LUSC: Lung squamous cell carcinoma
KIRC: Kidney renal clear cell carcinoma
GBM: Glioblastoma multiforme
SCC: Squamous cell carcinoma
MMP: Matrix metalloproteinase
TGFβ: Transforming growth factor β
SMAD: Drosophila mothers against decapentaplegic homolog
EMT: Epithelial mesenchymal transition
TNBC: Triple negative breast cancer
SC: Stem cell
TME: Tumor microenvironment
OSCC: Oral squamous cell carcinoma
FN: Fibronectin
FGFR1: Fibroblast growth factor receptor
PCa: Prostate cancer
RTK: Receptor tyrosine kinase
MCAs: Multi cellular aggregates
ZIKV: Zika virus
CRPC: Castration resistance prostate cancer
sEV: Small extracellular vesicle
GFR: Growth factor receptor
PDGF: Platelet derived growth factor
VEGF: Vascular endothelial growth factor
EGFR: Epidermal growth factor receptor
VM: Vasculogenic mimicry
MS: Multiple sclerosis
ADCC: Antibody dependent cellular cytotoxicity
PFS: Progression free survival
mCRC: Metastatic colorectal cancer
NSCLC: Non-small cell lung carcinoma
MGMT: Methylated O-6-methylguanine-DNA methyltransferase
GST: *Gleditsia sinensis* Thorns
RCP: Rab coupling protein
PET: Positron emission tomography
SPECT: Single photon emission computed tomography
siRNA: Small interfering RNA
HCC: Hepatocellular carcinoma
TRT: Targeted radionuclide therapy
CAR: Chimeric antigen receptors
MM: Multiple myeloma

## References

[1] Hynes RO (2002). Integrins: bidirectional, allosteric signaling machines. Cell.

[2] Su CY, Li JQ, Zhang LL, Wang H, Wang FH, Tao YW, Wang YQ, Guo QR, Li JJ, Liu Y, Yan YY, Zhang JY (2020). The biological functions and clinical applications of integrins in cancers. Front Pharmacol.

[3] Humphries JD, Byron A, Humphries MJ (2006). Integrin ligands at a glance. J Cell Sci.

[4] Cooper J, Giancotti FG (2019). Integrin signaling in cancer: mechanotransduction, stemness, epithelial plasticity, and therapeutic resistance. Cancer Cell.

[5] Larson RS, Corbi AL, Berman L, Springer T (1989). Primary structure of the leukocyte function-associated molecule-1 alpha subunit: an integrin with an embedded domain defining a protein superfamily. J Cell Biol.

[6] Calderwood DA, Tuckwell DS, Eble J, Kühn K, Humphries MJ (1997). The integrin alpha1 A-domain is a ligand binding site for collagens and laminin. J Biol Chem.

[7] Humphries MJ, Symonds EJ, Mould AP (2003). Mapping functional residues onto integrin crystal structures. Curr Opin Struct Biol.

[8] Kadry YA, Calderwood DA (2020). Chapter 22: structural and signaling functions of integrins. Biochim Biophys Acta Biomembr.

[9] Michael M, Parsons M (2020). New perspectives on integrin-dependent adhesions. Curr Opin Cell Biol.

[10] Kim C, Ye F, Ginsberg MH (2011). Regulation of integrin activation. Annu Rev Cell Dev Biol.

[11] Sun Z, Costell M, Fässler R (2019). Integrin activation by talin, kindlin and mechanical forces. Nat Cell Biol.

[12] Hamidi H, Ivaska J (2018). Every step of the way: integrins in cancer progression and metastasis. Nat Rev Cancer.

[13] Mainiero F, Murgia C, Wary KK, Curatola AM, Pepe A, Blumemberg M, Westwick JK, Der CJ, Giancotti FG (1997). The coupling of alpha6beta4 integrin to Ras-MAP kinase pathways mediated by Shc controls keratinocyte proliferation. Embo j.

[14] Shaw LM, Rabinovitz I, Wang HH, Toker A, Mercurio AM (1997). Activation of phosphoinositide 3-OH kinase by the alpha6beta4 integrin promotes carcinoma invasion. Cell.

[15] Raftopoulou M, Hall A (2004). Cell migration: Rho GTPases lead the way. Dev Biol.

[16] Aksorn N, Chanvorachote P (2019). Integrin as a molecular target for anti-cancer approaches in lung cancer. Anticancer Res.

[17] Wehrle-Haller B, Imhof BA (2003). Integrin-dependent pathologies. J Pathol.

[18] Hogg N, Bates PA (2000). Genetic analysis of integrin function in man: LAD-1 and other syndromes. Matrix Biol.

[19] Reynolds LE, Wyder L, Lively JC, Taverna D, Robinson SD, Huang X, Sheppard D, Hynes RO, Hodivala-Dilke KM (2002). Enhanced pathological angiogenesis in mice lacking beta3 integrin or beta3 and beta5 integrins. Nat Med.

[20] Guo W, Giancotti FG (2004). Integrin signalling during tumour progression. Nat Rev Mol Cell Biol.

[21] Cagnet S, Faraldo MM, Kreft M, Sonnenberg A, Raymond K, Glukhova MA (2013). Signaling events mediated by α3β1 integrin are essential for mammary tumorigenesis. Oncogene.

[22] Ramirez NE, Zhang Z, Madamanchi A, Boyd KL, O'Rear LD, Nashabi A, Li Z, Dupont WD, Zijlstra A, Zutter MM (2011). The alpha(2)beta(1) integrin is a metastasis suppressor in mouse models and human cancer. J Clin Invest.

[23] Gao J, Aksoy BA, Dogrusoz U, Dresdner G, Gross B, Sumer SO, Sun Y, Jacobsen A, Sinha R, Larsson E, Cerami E, Sander C, Schultz N (2013). Integrative analysis of complex cancer genomics and clinical profiles using the cBioPortal. Sci Signal.

[24] Niu J, Li Z (2017). The roles of integrin alphavbeta6 in cancer. Cancer Lett.

[25] Breuss JM, Gallo J, DeLisser HM, Klimanskaya IV, Folkesson HG, Pittet JF, Nishimura SL, Aldape K, Landers DV, Carpenter W (1995). Expression of the beta 6 integrin subunit in development, neoplasia and tissue repair suggests a role in epithelial remodeling. J Cell Sci.

[26] Katoh D, Nagaharu K, Shimojo N, Hanamura N, Yamashita M, Kozuka Y, Imanaka-Yoshida K, Yoshida T (2013). Binding of αvβ1 and αvβ6 integrins to tenascin-C induces epithelial-mesenchymal transition-like change of breast cancer cells. Oncogenesis.

[27] Kawashima A, Tsugawa S, Boku A, Kobayashi M, Minamoto T, Nakanishi I, Oda Y (2003). Expression of alphav integrin family in gastric carcinomas: increased alphavbeta6 is associated with lymph node metastasis. Pathol Res Pract.

[28] Lu H, Wang T, Li J, Fedele C, Liu Q, Zhang J, Jiang Z, Jain D, Iozzo RV, Violette SM, Weinreb PH, Davis RJ, Gioeli D, FitzGerald TJ, Altieri DC, Languino LR (2016). alphavbeta6 integrin promotes castrate-resistant prostate cancer through JNK1-mediated activation of androgen receptor. Cancer Res.

[29] Bates RC, Bellovin DI, Brown C, Maynard E, Wu B, Kawakatsu H, Sheppard D, Oettgen P, Mercurio AM (2005). Transcriptional activation of integrin beta6 during the epithelial-mesenchymal transition defines a novel prognostic indicator of aggressive colon carcinoma. J Clin Invest.

[30] Zhang ZY, Xu KS, Wang JS, Yang GY, Wang W, Wang JY, Niu WB, Liu EY, Mi YT, Niu J (2008). Integrin alphanvbeta6 acts as a prognostic indicator in gastric carcinoma. Clin Oncol (R Coll Radiol).

[31] Thomas GJ, Lewis MP, Hart IR, Marshall JF, Speight PM (2001). AlphaVbeta6 integrin promotes invasion of squamous carcinoma cells through up-regulation of matrix metalloproteinase-9. Int J Cancer.

[32] Liu S, Wang J, Niu W, Liu E, Wang J, Peng C, Lin P, Wang B, Khan AQ, Gao H, Liang B, Shahbaz M, Niu J (2013). The beta6-integrin-ERK/MAP kinase pathway contributes to chemo resistance in colon cancer. Cancer Lett.

[33] Margadant C, Sonnenberg A (2010). Integrin-TGF-beta crosstalk in fibrosis, cancer and wound healing. EMBO Rep.

[34] Bagati A, Kumar S, Jiang P, Pyrdol J, Zou AE, Godicelj A, Mathewson ND, Cartwright ANR, Cejas P, Brown M, Giobbie-Hurder A, Dillon D, Agudo J, Mittendorf EA, Liu XS, Wucherpfennig KW (2021). Integrin alphavbeta6-TGFbeta-SOX4 pathway drives immune evasion in triple-negative breast cancer. Cancer Cell.

[35] Kanda R, Kawahara A, Watari K, Murakami Y, Sonoda K, Maeda M, Fujita H, Kage M, Uramoto H, Costa C, Kuwano M, Ono M (2013). Erlotinib resistance in lung cancer cells mediated by integrin β1/Src/Akt-driven bypass signaling. Cancer Res.

[36] Sottnik JL, Daignault-Newton S, Zhang X, Morrissey C, Hussain MH, Keller ET, Hall CL (2013). Integrin alpha2beta 1 (α2β1) promotes prostate cancer skeletal metastasis. Clin Exp Metastasis.

[37] Bartolomé RA, Barderas R, Torres S, Fernandez-Aceñero MJ, Mendes M, García-Foncillas J, Lopez-Lucendo M, Casal JI (2014). Cadherin-17 interacts with α2β1 integrin to regulate cell proliferation and adhesion in colorectal cancer cells causing liver metastasis. Oncogene.

[38] Varzavand A, Hacker W, Ma D, Gibson-Corley K, Hawayek M, Tayh OJ, Brown JA, Henry MD, Stipp CS (2016). alpha3beta1 integrin suppresses prostate cancer metastasis via regulation of the hippo pathway. Cancer Res.

[39] Hu T, Zhou R, Zhao Y, Wu G (2016). Integrin α6/Akt/Erk signaling is essential for human breast cancer resistance to radiotherapy. Sci Rep.

[40] Haas TL, Sciuto MR, Brunetto L, Valvo C, Signore M, Fiori ME, di Martino S, Giannetti S, Morgante L, Boe A, Patrizii M, Warnken U, Schnölzer M, Ciolfi A, Di Stefano C, Biffoni M, Ricci-Vitiani L, Pallini R, De Maria R (2017). Integrin α7 is a functional marker and potential therapeutic target in glioblastoma. Cell Stem Cell.

[41] Gupta SK, Oommen S, Aubry MC, Williams BP, Vlahakis NE (2013). Integrin α9β1 promotes malignant tumor growth and metastasis by potentiating epithelial-mesenchymal transition. Oncogene.

[42] Munksgaard Thorén M, Chmielarska Masoumi K, Krona C, Huang X, Kundu S, Schmidt L, Forsberg-Nilsson K, Floyd Keep M, Englund E, Nelander S, Holmqvist B, Lundgren-Åkerlund E (2019). Integrin α10, a novel therapeutic target in glioblastoma, regulates cell migration, proliferation, and survival. Cancers (Basel).

[43] Salvo E, Garasa S, Dotor J, Morales X, Pelaez R, Altevogt P, Rouzaut A (2014). Combined targeting of TGF-beta1 and integrin beta3 impairs lymph node metastasis in a mouse model of non-small-cell lung cancer. Mol Cancer.

[44] Fu Y, Zhang Y, Lei Z, Liu T, Cai T, Wang A, Du W, Zeng Y, Zhu J, Liu Z, Huang JA (2020). Abnormally activated OPN/integrin alphaVbeta3/FAK signalling is responsible for EGFR-TKI resistance in EGFR mutant non-small-cell lung cancer. J Hematol Oncol.

[45] Wang T, Huang J, Vue M, Alavian MR, Goel HL, Altieri DC, Languino LR, FitzGerald TJ (2019). alphavbeta3 integrin mediates radioresistance of prostate cancer cells through regulation of survivin. Mol Cancer Res.

[46] Seguin L, Kato S, Franovic A, Camargo MF, Lesperance J, Elliott KC, Yebra M, Mielgo A, Lowy AM, Husain H, Cascone T, Diao L, Wang J, Wistuba II, Heymach JV, Lippman SM, Desgrosellier JS, Anand S, Weis SM, Cheresh DA (2014). An integrin beta(3)-KRAS-RalB complex drives tumour stemness and resistance to EGFR inhibition. Nat Cell Biol.

[47] Sung JS, Kang CW, Kang S, Jang Y, Chae YC, Kim BG, Cho NH (2020). ITGB4-mediated metabolic reprogramming of cancer-associated fibroblasts. Oncogene.

[48] Jin S, Lee WC, Aust D, Pilarsky C, Cordes N (2019). β8 integrin mediates pancreatic cancer cell radiochemoresistance. Mol Cancer Res.

[49] Zhang D, Tang DG (2018). "Splice" a way towards neuroendocrine prostate cancer. EBioMedicine.

[50] Singh A, Settleman J (2010). EMT, cancer stem cells and drug resistance: an emerging axis of evil in the war on cancer. Oncogene.

[51] Zhang D, Tang DG, Rycaj K (2018). Cancer stem cells: regulation programs, immunological properties and immunotherapy. Semin Cancer Biol.

[52] Tang DG (2012). Understanding cancer stem cell heterogeneity and plasticity. Cell Res.

[53] Seguin L, Desgrosellier JS, Weis SM, Cheresh DA (2015). Integrins and cancer: regulators of cancer stemness, metastasis, and drug resistance. Trends Cell Biol.

[54] Lathia JD, Gallagher J, Heddleston JM, Wang J, Eyler CE, Macswords J, Wu Q, Vasanji A, McLendon RE, Hjelmeland AB, Rich JN (2010). Integrin alpha 6 regulates glioblastoma stem cells. Cell Stem Cell.

[55] Lo PK, Kanojia D, Liu X, Singh UP, Berger FG, Wang Q, Chen H (2012). CD49f and CD61 identify Her2/neu-induced mammary tumor-initiating cells that are potentially derived from luminal progenitors and maintained by the integrin-TGFβ signaling. Oncogene.

[56] Ming XY, Fu L, Zhang LY, Qin YR, Cao TT, Chan KW, Ma S, Xie D, Guan XY (2016). Integrin alpha7 is a functional cancer stem cell surface marker in oesophageal squamous cell carcinoma. Nat Commun.

[57] Jahangiri A, Aghi MK, Carbonell WS (2014). beta1 integrin: Critical path to antiangiogenic therapy resistance and beyond. Cancer Res.

[58] Schober M, Fuchs E (2011). Tumor-initiating stem cells of squamous cell carcinomas and their control by TGF-beta and integrin/focal adhesion kinase (FAK) signaling. Proc Natl Acad Sci U S A.

[59] Truong HH, Xiong J, Ghotra VP, Nirmala E, Haazen L, Le-Devedec SE, Balcioglu HE, He S, Snaar-Jagalska BE, Vreugdenhil E, Meerman JH, van de Water B, Danen EH: Beta1 integrin inhibition elicits a prometastatic switch through the TGFbeta-miR-200-ZEB network in E-cadherin-positive triple-negative breast cancer. *Sci Signal* 2014, 7(312): ra15. 10.1126/scisignal.2004751 24518294

[60] Kawahara R, Niwa Y, Simizu S (2018). Integrin beta1 is an essential factor in vasculogenic mimicry of human cancer cells. Cancer Sci.

[61] Kowalski-Chauvel A, Gouaze-Andersson V, Baricault L, Martin E, Delmas C, Toulas C, Cohen-Jonathan-Moyal E, Seva C (2019). Alpha6-integrin regulates FGFR1 expression through the ZEB1/YAP1 transcription complex in glioblastoma stem cells resulting in enhanced proliferation and stemness. Cancers (Basel).

[62] Goel HL, Gritsko T, Pursell B, Chang C, Shultz LD, Greiner DL, Norum JH, Toftgard R, Shaw LM, Mercurio AM (2014). Regulated splicing of the alpha6 integrin cytoplasmic domain determines the fate of breast cancer stem cells. Cell Rep.

[63] Goel HL, Pursell B, Chang C, Shaw LM, Mao J, Simin K, Kumar P, Vander Kooi CW, Shultz LD, Greiner DL, Norum JH, Toftgard R, Kuperwasser C, Mercurio AM (2013). GLI1 regulates a novel neuropilin-2/α6β1 integrin based autocrine pathway that contributes to breast cancer initiation. EMBO Mol Med.

[64] Zoni E, van der Horst G, van de Merbel AF, Chen L, Rane JK, Pelger RC, Collins AT, Visakorpi T, Snaar-Jagalska BE, Maitland NJ, van der Pluijm G (2015). miR-25 modulates invasiveness and dissemination of human prostate cancer cells via regulation of alphav- and alpha6-integrin expression. Cancer Res.

[65] Boger C, Warneke VS, Behrens HM, Kalthoff H, Goodman SL, Becker T, Rocken C (2015). Integrins alphavbeta3 and alphavbeta5 as prognostic, diagnostic, and therapeutic targets in gastric cancer. Gastric Cancer.

[66] Alday-Parejo B, Stupp R, Ruegg C (2019). Are integrins still practicable targets for anti-cancer therapy?. Cancers (Basel).

[67] Dong H, Liu H, Zhou W, Zhang F, Li C, Chen J, Tan C, Tang B, Yu P (2019). GLI1 activation by non-classical pathway integrin alphavbeta3/ERK1/2 maintains stem cell-like phenotype of multicellular aggregates in gastric cancer peritoneal metastasis. Cell Death Dis.

[68] Monnier Y, Farmer P, Bieler G, Imaizumi N, Sengstag T, Alghisi GC, Stehle JC, Ciarloni L, Andrejevic-Blant S, Moeckli R, Mirimanoff RO, Goodman SL, Delorenzi M, Ruegg C (2008). CYR61 and alphaVbeta5 integrin cooperate to promote invasion and metastasis of tumors growing in preirradiated stroma. Cancer Res.

[69] Berghoff AS, Kovanda AK, Melchardt T, Bartsch R, Hainfellner JA, Sipos B, Schittenhelm J, Zielinski CC, Widhalm G, Dieckmann K, Weller M, Goodman SL, Birner P, Preusser M (2014). alphavbeta3, alphavbeta5 and alphavbeta6 integrins in brain metastases of lung cancer. Clin Exp Metastasis.

[70] Zhu Z, Mesci P, Bernatchez JA, Gimple RC, Wang X, Schafer ST, Wettersten HI, Beck S, Clark AE, Wu Q, Prager BC, Kim LJY, Dhanwani R, Sharma S, Garancher A, Weis SM, Mack SC, Negraes PD, Trujillo CA, Penalva LO, Feng J, Lan Z, Zhang R, Wessel AW, Dhawan S, Diamond MS, Chen CC, Wechsler-Reya RJ, Gage FH, Hu H (2020). Zika Virus Targets Glioblastoma Stem Cells through a SOX2-Integrin alphavbeta5 Axis. Cell Stem Cell.

[71] Malric L, Monferran S, Delmas C, Arnauduc F, Dahan P, Boyrie S, Deshors P, Lubrano V, Da Mota DF, Gilhodes J, Filleron T, Siegfried A, Evrard S, Kowalski-Chauvel A, Moyal EC-J, Toulas C, Lemarié A. Inhibiting integrin β8 to differentiate and radiosensitize glioblastoma-initiating cells. Mol Cancer Res 2019;17(2):384–97. 10.1158/1541-7786.MCR-18-0386 30266751

[72] Guerrero PA, Tchaicha JH, Chen Z, Morales JE, McCarty N, Wang Q, Sulman EP, Fuller G, Lang FF, Rao G, McCarty JH (2017). Glioblastoma stem cells exploit the alphavbeta8 integrin-TGFbeta1 signaling axis to drive tumor initiation and progression. Oncogene.

[73] Casari I, Howard JA, Robless EE, Falasca M (2021). Exosomal integrins and their influence on pancreatic cancer progression and metastasis. Cancer Lett.

[74] Adem B, Vieira PF, Melo SA (2020). Decoding the Biology of Exosomes in Metastasis. Trends Cancer.

[75] Fedele C, Singh A, Zerlanko BJ, Iozzo RV, Languino LR (2015). The αvβ6 integrin is transferred intercellularly via exosomes. J Biol Chem.

[76] Quaglia F, Krishn SR, Daaboul GG, Sarker S, Pippa R, Domingo-Domenech J, Kumar G, Fortina P, McCue P, Kelly WK, Beltran H, Liu Q, Languino LR (2020). Small extracellular vesicles modulated by αVβ3 integrin induce neuroendocrine differentiation in recipient cancer cells. J Extracell Vesicles.

[77] Hoshino A, Costa-Silva B, Shen TL, Rodrigues G, Hashimoto A, Tesic Mark M, Molina H, Kohsaka S, Di Giannatale A, Ceder S, Singh S, Williams C, Soplop N, Uryu K, Pharmer L, King T, Bojmar L, Davies AE, Ararso Y, Zhang T, Zhang H, Hernandez J, Weiss JM, Dumont-Cole VD, Kramer K, Wexler LH, Narendran A, Schwartz GK, Healey JH, Sandstrom P (2015). Tumour exosome integrins determine organotropic metastasis. Nature.

[78] Bierie B, Pierce SE, Kroeger C, Stover DG, Pattabiraman DR, Thiru P, Liu Donaher J, Reinhardt F, Chaffer CL, Keckesova Z, Weinberg RA (2017). Integrin-β4 identifies cancer stem cell-enriched populations of partially mesenchymal carcinoma cells. Proc Natl Acad Sci U S A.

[79] Masugi Y, Yamazaki K, Emoto K, Effendi K, Tsujikawa H, Kitago M, Itano O, Kitagawa Y, Sakamoto M (2015). Upregulation of integrin β4 promotes epithelial-mesenchymal transition and is a novel prognostic marker in pancreatic ductal adenocarcinoma. Lab Invest.

[80] Yoshioka T, Otero J, Chen Y, Kim YM, Koutcher JA, Satagopan J, Reuter V, Carver B, de Stanchina E, Enomoto K, Greenberg NM, Scardino PT, Scher HI, Sawyers CL, Giancotti FG (2013). β4 Integrin signaling induces expansion of prostate tumor progenitors. J Clin Invest.

[81] Vaillant F, Asselin-Labat ML, Shackleton M, Forrest NC, Lindeman GJ, Visvader JE (2008). The mammary progenitor marker CD61/beta3 integrin identifies cancer stem cells in mouse models of mammary tumorigenesis. Cancer Res.

[82] Malric L, Monferran S, Delmas C, Arnauduc F, Dahan P, Boyrie S, Deshors P, Lubrano V, Da Mota DF, Gilhodes J, Filleron T, Siegfried A, Evrard S, Kowalski-Chauvel A, Moyal EC, Toulas C, Lemarié A (2019). Inhibiting Integrin β8 to Differentiate and Radiosensitize Glioblastoma-Initiating Cells. Mol Cancer Res.

[83] Desgrosellier JS, Lesperance J, Seguin L, Gozo M, Kato S, Franovic A, Yebra M, Shattil SJ, Cheresh DA (2014). Integrin αvβ3 drives slug activation and stemness in the pregnant and neoplastic mammary gland. Dev Cell.

[84] Huang W, Yan Y, Liu Y, Lin M, Ma J, Zhang W, Dai J, Li J, Guo Q, Chen H, Makabel B, Liu H, Su C, Bi H, Zhang J (2020). Exosomes with low miR-34c-3p expression promote invasion and migration of non-small cell lung cancer by upregulating integrin α2β1. Signal Transduct Target Ther.

[85] Wu X, Cai J, Zuo Z, Li J (2019). Collagen facilitates the colorectal cancer stemness and metastasis through an integrin/PI3K/AKT/Snail signaling pathway. Biomed Pharmacother.

[86] Moon JH, Rho YS, Lee SH, Koo BS, Lee HJ, Do SI, Cho JH, Eun YG, Park MW, Shin HA, Lim YC (2019). Role of integrin β1 as a biomarker of stemness in head and neck squamous cell carcinoma. Oral Oncol.

[87] Lin HC, Wu CL, Chen YL, Huang JS, Wong TY, Yuan K (2014). High-level β1-integrin expression in a subpopulation of highly tumorigenic oral cancer cells. Clin Oral Investig.

[88] Ley K, Rivera-Nieves J, Sandborn WJ, Shattil S (2016). Integrin-based therapeutics: biological basis, clinical use and new drugs. Nat Rev Drug Discov.

[89] Estevez B, Shen B, Du X (2015). Targeting integrin and integrin signaling in treating thrombosis. Arterioscler Thromb Vasc Biol.

[90] González-Suarez I, de Antonio LR, Orviz A, Moreno-García S, Valle-Arcos MD, Matias-Guiu JA, Valencia C, Jorquera Moya M, Oreja-Guevara C (2017). Catastrophic outcome of patients with a rebound after Natalizumab treatment discontinuation. Brain Behav.

[91] Chen Q, Manning CD, Millar H, McCabe FL, Ferrante C, Sharp C, Shahied-Arruda L, Doshi P, Nakada MT, Anderson GM (2008). CNTO 95, a fully human anti alphav integrin antibody, inhibits cell signaling, migration, invasion, and spontaneous metastasis of human breast cancer cells. Clin Exp Metastasis.

[92] Mullamitha SA, Ton NC, Parker GJ, Jackson A, Julyan PJ, Roberts C, Buonaccorsi GA, Watson Y, Davies K, Cheung S, Hope L, Valle JW, Radford JA, Lawrance J, Saunders MP, Munteanu MC, Nakada MT, Nemeth JA, Davis HM, Jiao Q, Prabhakar U, Lang Z, Corringham RE, Beckman RA, Jayson GC (2007). Phase I evaluation of a fully human anti-alphav integrin monoclonal antibody (CNTO 95) in patients with advanced solid tumors. Clin Cancer Res.

[93] O'Day S, Pavlick A, Loquai C, Lawson D, Gutzmer R, Richards J, Schadendorf D, Thompson JA, Gonzalez R, Trefzer U, Mohr P, Ottensmeier C, Chao D, Zhong B, de Boer CJ, Uhlar C, Marshall D, Gore ME, Lang Z, Hait W, Ho P (2011). A randomised, phase II study of intetumumab, an anti-αv-integrin mAb, alone and with dacarbazine in stage IV melanoma. Br J Cancer.

[94] Hussain M, Le Moulec S, Gimmi C, Bruns R, Straub J, Miller K (2016). Differential effect on bone lesions of targeting integrins: randomized phase II trial of abituzumab in patients with metastatic castration-resistant prostate cancer. Clin Cancer Res.

[95] Élez E, Kocáková I, Höhler T, Martens UM, Bokemeyer C, Van Cutsem E, Melichar B, Smakal M, Csőszi T, Topuzov E, Orlova R, Tjulandin S, Rivera F, Straub J, Bruns R, Quaratino S, Tabernero J (2015). Abituzumab combined with cetuximab plus irinotecan versus cetuximab plus irinotecan alone for patients with KRAS wild-type metastatic colorectal cancer: the randomised phase I/II POSEIDON trial. Ann Oncol.

[96] Brooks PC, Stromblad S, Klemke R, Visscher D, Sarkar FH, Cheresh DA (1995). Antiintegrin alpha v beta 3 blocks human breast cancer growth and angiogenesis in human skin. J Clin Invest.

[97] Mitjans F, Meyer T, Fittschen C, Goodman S, Jonczyk A, Marshall JF, Reyes G, Piulats J (2000). In vivo therapy of malignant melanoma by means of antagonists of alphav integrins. Int J Cancer.

[98] Hersey P, Sosman J, O'Day S, Richards J, Bedikian A, Gonzalez R, Sharfman W, Weber R, Logan T, Buzoianu M, Hammershaimb L, Kirkwood JM (2010). A randomized phase 2 study of etaracizumab, a monoclonal antibody against integrin alpha(v)beta(3), + or - dacarbazine in patients with stage IV metastatic melanoma. Cancer.

[99] Bhaskar V, Zhang D, Fox M, Seto P, Wong MH, Wales PE, Powers D, Chao DT, Dubridge RB, Ramakrishnan V (2007). A function blocking anti-mouse integrin alpha5beta1 antibody inhibits angiogenesis and impedes tumor growth in vivo. J Transl Med.

[100] Bell-McGuinn KM, Matthews CM, Ho SN, Barve M, Gilbert L, Penson RT, Lengyel E, Palaparthy R, Gilder K, Vassos A, McAuliffe W, Weymer S, Barton J, Schilder RJ (2011). A phase II, single-arm study of the anti-α5β1 integrin antibody volociximab as monotherapy in patients with platinum-resistant advanced epithelial ovarian or primary peritoneal cancer. Gynecol Oncol.

[101] Almokadem S, Belani CP (2012). Volociximab in cancer. Expert Opin Biol Ther.

[102] Mas-Moruno C, Rechenmacher F, Kessler H (2010). Cilengitide: the first anti-angiogenic small molecule drug candidate design, synthesis and clinical evaluation. Anticancer Agents Med Chem.

[103] Reardon DA, Fink KL, Mikkelsen T, Cloughesy TF, O'Neill A, Plotkin S, Glantz M, Ravin P, Raizer JJ, Rich KM, Schiff D, Shapiro WR, Burdette-Radoux S, Dropcho EJ, Wittemer SM, Nippgen J, Picard M, Nabors LB (2008). Randomized phase II study of cilengitide, an integrin-targeting arginine-glycine-aspartic acid peptide, in recurrent glioblastoma multiforme. J Clin Oncol.

[104] Stupp R, Hegi ME, Gorlia T, Erridge SC, Perry J, Hong Y-K, Aldape KD, Lhermitte B, Pietsch T, Grujicic D, Steinbach JP, Wick W, Tarnawski R, Nam D-H, Hau P, Weyerbrock A, Taphoorn MJB, Shen C-C, Rao N, Thurzo L, Herrlinger U, Gupta T, Kortmann R-D, Adamska K, McBain C, Brandes AA, Tonn JC, Schnell O, Wiegel T, Kim C-Y (2014). Cilengitide combined with standard treatment for patients with newly diagnosed glioblastoma with methylated MGMT promoter (CENTRIC EORTC 26071–22072 study): a multicentre, randomised, open-label, phase 3 trial. Lancet Oncol.

[105] Cianfrocca ME, Kimmel KA, Gallo J, Cardoso T, Brown MM, Hudes G, Lewis N, Weiner L, Lam GN, Brown SC, Shaw DE, Mazar AP, Cohen RB (2006). Phase 1 trial of the antiangiogenic peptide ATN-161 (Ac-PHSCN-NH(2)), a beta integrin antagonist, in patients with solid tumours. Br J Cancer.

[106] Semba T, Funahashi Y, Ono N, Yamamoto Y, Sugi NH, Asada M, Yoshimatsu K, Wakabayashi T (2004). An angiogenesis inhibitor E7820 shows broad-spectrum tumor growth inhibition in a xenograft model: possible value of integrin alpha2 on platelets as a biological marker. Clin Cancer Res.

[107] Sawyer MB, Iq Ba LS, Lenz H, Lima C, Rossignol DP, Krivelevich I, Fan J, El-Khoueiry AB (2010). Phase II study of E7820 in combination with cetuximab in subjects (pts) with metastatic and refractory colorectal cancer (CRC). J Clin Oncol.

[108] Cirkel GA, Kerklaan BM, Vanhoutte F, Van der Aa A, Lorenzon G, Namour F, Pujuguet P, Darquenne S, de Vos FY, Snijders TJ, Voest EE, Schellens JH, Lolkema MP (2016). A dose escalating phase I study of GLPG0187, a broad spectrum integrin receptor antagonist, in adult patients with progressive high-grade glioma and other advanced solid malignancies. Invest New Drugs.

[109] Rosenthal MA, Davidson P, Rolland F, Campone M, Xue L, Han TH, Mehta A, Berd Y, He W, Lombardi A (2010). Evaluation of the safety, pharmacokinetics and treatment effects of an alpha(nu)beta(3) integrin inhibitor on bone turnover and disease activity in men with hormone-refractory prostate cancer and bone metastases. Asia Pac J Clin Oncol.

[110] Ryu S, Park KM, Lee SH (2016). Gleditsia sinensis Thorn Attenuates the Collagen-based migration of PC3 prostate cancer cells through the suppression of α2β1 integrin expression. Int J Mol Sci.

[111] Lin TH, Tan TW, Tsai TH, Chen CC, Hsieh TF, Lee SS, Liu HH, Chen WC, Tang CH (2013). D-pinitol inhibits prostate cancer metastasis through inhibition of αVβ3 integrin by modulating FAK, c-Src and NF-κB pathways. Int J Mol Sci.

[112] Kotha RR, Luthria DL. Curcumin: biological, pharmaceutical, nutraceutical, and analytical aspects. Molecules 2019, 24(16). 10.3390/molecules24162930 PMC6720683 31412624

[113] Gupta SC, Patchva S, Aggarwal BB (2013). Therapeutic roles of curcumin: lessons learned from clinical trials. Aaps J.

[114] Choe SR, Kim YN, Park CG, Cho KH, Cho DY, Lee HY (2018). RCP induces FAK phosphorylation and ovarian cancer cell invasion with inhibition by curcumin. Exp Mol Med.

[115] Chen X, Hou Y, Tohme M, Park R, Khankaldyyan V, Gonzales-Gomez I, Bading JR, Laug WE, Conti PS (2004). Pegylated Arg-Gly-Asp peptide: 64Cu labeling and PET imaging of brain tumor alphavbeta3-integrin expression. J Nucl Med.

[116] Gaertner FC, Kessler H, Wester HJ, Schwaiger M, Beer AJ (2012). Radiolabelled RGD peptides for imaging and therapy. Eur J Nucl Med Mol Imaging.

[117] Sharma R, Valls PO, Inglese M, Dubash S, Chen M, Gabra H, Montes A, Challapalli A, Arshad M, Tharakan G, Chambers E, Cole T, Lozano-Kuehne JP, Barwick TD, Aboagye EO (2020). [(18)F]Fluciclatide PET as a biomarker of response to combination therapy of pazopanib and paclitaxel in platinum-resistant/refractory ovarian cancer. Eur J Nucl Med Mol Imaging.

[118] Eo JS, Jeong JM (2016). Angiogenesis Imaging Using (68)Ga-RGD PET/CT: Therapeutic Implications. Semin Nucl Med.

[119] Murphy EA, Majeti BK, Barnes LA, Makale M, Weis SM, Lutu-Fuga K, Wrasidlo W, Cheresh DA (2008). Nanoparticle-mediated drug delivery to tumor vasculature suppresses metastasis. Proc Natl Acad Sci USA.

[120] Bogorad RL, Yin H, Zeigerer A, Nonaka H, Ruda VM, Zerial M, Anderson DG, Koteliansky V (2014). Nanoparticle-formulated siRNA targeting integrins inhibits hepatocellular carcinoma progression in mice. Nat Commun.

[121] Heidenreich A, Rawal SK, Szkarlat K, Bogdanova N, Dirix L, Stenzl A, Welslau M, Wang G, Dawkins F, de Boer CJ, Schrijvers D (2013). A randomized, double-blind, multicenter, phase 2 study of a human monoclonal antibody to human αν integrins (intetumumab) in combination with docetaxel and prednisone for the first-line treatment of patients with metastatic castration-resistant prostate cancer. Ann Oncol.

[122] Vermorken JB, Peyrade F, Krauss J, Mesía R, Remenar E, Gauler TC, Keilholz U, Delord JP, Schafhausen P, Erfán J, Brümmendorf TH, Iglesias L, Bethe U, Hicking C, Clement PM (2014). Cisplatin, 5-fluorouracil, and cetuximab (PFE) with or without cilengitide in recurrent/metastatic squamous cell carcinoma of the head and neck: results of the randomized phase I/II ADVANTAGE trial (phase II part). Ann Oncol.

[123] Chen H, Zhao L, Fu K, Lin Q, Wen X, Jacobson O, Sun L, Wu H, Zhang X, Guo Z, Lin Q, Chen X (2019). Integrin α(v)β(3)-targeted radionuclide therapy combined with immune checkpoint blockade immunotherapy synergistically enhances anti-tumor efficacy. Theranostics.

[124] Ruan S, Lin M, Zhu Y, Lum L, Thakur A, Jin R, Shao W, Zhang Y, Hu Y, Huang S, Hurt EM, Chang AE, Wicha MS, Li Q (2020). Integrin β4-targeted cancer immunotherapies inhibit tumor growth and decrease metastasis. Cancer Res.

[125] Kwan BH, Zhu EF, Tzeng A, Sugito HR, Eltahir AA, Ma B, Delaney MK, Murphy PA, Kauke MJ, Angelini A, Momin N, Mehta NK, Maragh AM, Hynes RO, Dranoff G, Cochran JR, Wittrup KD (2017). Integrin-targeted cancer immunotherapy elicits protective adaptive immune responses. J Exp Med.

[126] Hosen N, Matsunaga Y, Hasegawa K, Matsuno H, Nakamura Y, Makita M, Watanabe K, Yoshida M, Satoh K, Morimoto S, Fujiki F, Nakajima H, Nakata J, Nishida S, Tsuboi A, Oka Y, Manabe M, Ichihara H, Aoyama Y, Mugitani A, Nakao T, Hino M, Uchibori R, Ozawa K, Baba Y, Terakura S, Wada N, Morii E, Nishimura J, Takeda K (2017). The activated conformation of integrin β(7) is a novel multiple myeloma-specific target for CAR T cell therapy. Nat Med.

